# Afforestation or intense pasturing improve the ecological and economic value of abandoned tropical farmlands

**DOI:** 10.1038/ncomms6612

**Published:** 2014-11-26

**Authors:** Thomas Knoke, Jörg Bendix, Perdita Pohle, Ute Hamer, Patrick Hildebrandt, Kristin Roos, Andrés Gerique, María L. Sandoval, Lutz Breuer, Alexander Tischer, Brenner Silva, Baltazar Calvas, Nikolay Aguirre, Luz M. Castro, David Windhorst, Michael Weber, Bernd Stimm, Sven Günter, Ximena Palomeque, Julio Mora, Reinhard Mosandl, Erwin Beck

**Affiliations:** 1 TUM School of Life Sciences Weihenstephan, Technische Universität München, 85354 Freising, Germany; 2 Laboratory for Climatology and Remote Sensing (LCRS), Faculty of Geography, University of Marburg, 35032 Marburg, Germany; 3 Institute of Geography, University of Erlangen-Nürnberg, 91058 Erlangen, Germany; 4 Institute of Soil Science and Site Ecology, Dresden University of Technology, 01737 Tharandt, Germany; 5 Department of Plant Physiology and Bayreuth Centre of Ecology and Environmental Research, University of Bayreuth, 95440 Bayreuth, Germany; 6 Departamento de Desarrollo Ambiente y territorio, Facultad Latinoamericana de Ciencias Sociales, FLACSO, 170516 Quito, Ecuador; 7 Institute for Landscape Ecology and Resources Management, Justus Liebig University Giessen, 35392 Giessen, Germany; 8 Biodiversity, Forestry and Ecosystem Services Research Program, National University of Loja, 110101 Loja, Ecuador; 9 Departamento de Economía, Universidad Técnica Particular de Loja, 1101608 Loja, Ecuador; 10 Tropical Agricultural Research and Higher Education Center (CATIE), 7170 Turrialba-Cartago, Costa Rica

## Abstract

Increasing demands for livelihood resources in tropical rural areas have led to progressive clearing of biodiverse natural forests. Restoration of abandoned farmlands could counter this process. However, as aims and modes of restoration differ in their ecological and socio-economic value, the assessment of achievable ecosystem functions and benefits requires holistic investigation. Here we combine the results from multidisciplinary research for a unique assessment based on a normalization of 23 ecological, economic and social indicators for four restoration options in the tropical Andes of Ecuador. A comparison of the outcomes among afforestation with native alder or exotic pine, pasture restoration with either low-input or intense management and the abandoned *status quo* shows that both variants of afforestation and intense pasture use improve the ecological value, but low-input pasture does not. Economic indicators favour either afforestation or intense pasturing. Both Mestizo and indigenous Saraguro settlers are more inclined to opt for afforestation.

## Abstract (web summary)

Restoration of abandoned farmlands has the potential to improve ecosystem functions and benefits. Using a multidisciplinary approach, Knoke *et al*. show that ecological and economic indicators favour either afforestation or intense pasturing of these abandoned areas in the tropical Andes.

## Graphical abstract

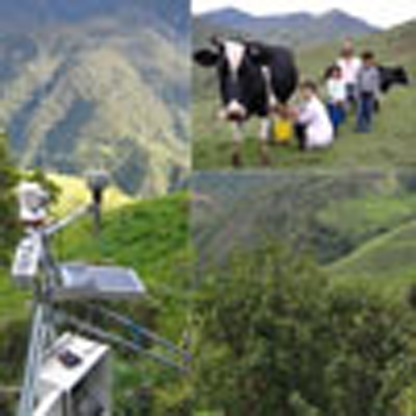

Decreasing agricultural yields and increasing national and global competition^1^,^2^ force farmers to abandon less productive lands and either clear more pristine forest for agriculture or give up agriculture as the basis of their livelihoods. Reclaiming abandoned areas to resume production is rarely considered a worthwhile alternative. Field *et al*.^3^ estimated that 386 M ha of abandoned lands worldwide have the potential for renewed productive use.

Re-utilization could not only mitigate the increasing pressures on natural forest, but could also help to alleviate poverty by improving food security^4^, to promote rural socio-economic development^5^ and to lower rural outmigration. Sustainability research must, therefore, investigate strategies to recover abandoned lands^6^.

Existing approaches to the problem of restoration have largely been focused on afforestation attempts and either their ecological^7^, economic^8^ or social^9^ consequences. A more holistic, but thus far unrealized, ideal of research into the benefits humans receive from ecosystems should provide biophysically realistic ecosystem data and models, consider local trade-offs, recognize off-site effects and involve stakeholders^10^. Moreover, to promote sustainable future land use, not only afforestation but also restoration of agricultural potential should be considered. Consequently, scientifically responsible decision support requires multidisciplinary long-term research that supports reliable parameterization of customized models.

Although benefit-specific ecosystem services are generally narrowly defined as components of nature that are directly enjoyed, consumed or used as final products and services^11^, we use a broader approach to assess the capacity of natural processes and components of restoration options to provide goods and services^12^. Our ecological indicators thus quantify ecosystem functions. Following the classification by Boyd and Banzhaf^11^, our socio-economic indicators are estimates of the benefits farmers may obtain from each restoration option.

In the tropical Andes of southern Ecuador, clearing of natural forest commonly follows the abandonment of pastures and thus represents a widespread example of unsustainable land use^13^,^14^. This practice occurs mostly in tropical mountain regions beginning at 1,500 m altitude and continuing up to the tree line^15^,^16^,^17^, in Latin America and also elsewhere^18^,^19^. In our study area, abandoned pastures have already grown to 35% of the total pasture area^20^. One major reason for this adverse development is the invasion of weeds—mainly tropical bracken fern, which is resistant to burning—the most common local weed control tool^21^. The use of fire begins with the clearing of the natural forest and is regularly applied thereafter for weed control and pasture rejuvenation. In this topographically diverse landscape with highly fragmented vegetation, productive alternatives to leaving areas abandoned are of utmost ecological and socio-economic importance^22^. This applies particularly to southern Ecuador where the native mountain forests contribute significantly to the outstanding biodiversity^23^ (Supplementary Methods). In the present work, we evaluate four different options for reintegrating abandoned pastures into the production process. The results of experiments—some running as long as 15 years—show that both afforestation^13^ and restoration of pasture^24^ (‘repasturization’) are feasible alternatives to leaving land abandoned. However, these results also suggest that large financial inputs as compared with the business-as-usual (BAU) option—pasturing after clearing of natural forest—are necessary to establish the restoration options.

The use of appropriate indicators is pivotal to answering policy-relevant questions concerning the potential benefits that people may obtain from ecosystems^25^. The establishment of standardized methods allows comparisons of ecosystem functions and benefits if they are adjusted for location and to address specific problems. However, the integration of multiple functions and benefits into a general assessment is still problematic and are relatively uncommon in the literature^25^. Our novel evaluation approach to quantifying and assessing the ecosystem functions and benefits of different land-use options is an attempt to solve these problems. It uses normalized indicators to make various ecosystem functions and benefits comparable, which, in this study, proves itself to be a robust method even under rigorous sensitivity assessments. We show that averaged ecological and socio-economic indicators are highly positively correlated. Afforestation ranks highest both from the ecological and the socio-economic points of view, followed by repasturization with subsequent intense pasturing. However, the options for land restoration provide relatively low short-term socio-economic benefits for farmers when compared with the BAU land use (pasturing after forest clearing). Because of this, to successfully promote restoration options as a way to relieve the pressure on biodiverse natural forests, a compensation amount of up to US$ 180 ha^−1^ per year may be necessary.

## Results

### Assessing ecosystem functions and benefits

We will first present our approach for assessing multiple ecosystem functions and benefits of five land-use options (Table 1). Next, we justify the selected indicators and briefly illustrate the process of assessing the various restoration options using data from our study area. Each indictor subsection concludes with highlighting the results of general importance for that indicator.

We use 23 indicators to characterize four key elements of ‘Ecological Functions’ and four key elements of ‘Socio-economic Benefits’ (Table 2), to thoroughly assess the potential ecosystem functions and benefits provided by the land-use options investigated. The indicators include supporting (biomass production and soil quality) and regulating functions (carbon, climate and hydrology), as well as provisioning (timber and food) and social benefits (acceptance by the local people), and are meant to represent a comprehensive set of indicators of the potential capacity for the various restoration options to generate benefits from ecosystems^26^.

Social acceptance serves here as an indicator of the cultural benefit, for example, the compatibility with traditional livelihoods, as well as their contribution to landscape aesthetics or preserving cultural heritage. Although people often consider both provisioning and regulating functions when expressing their preferences, they also tend to include intangible values of land use, which are largely determined by tradition, experience and personal preference. However, as intangible cultural values are impossible to measure in ecological units, social acceptance can be used as a meaningful proxy for cultural ecosystem benefits, which are often ignored in existing approaches to assessing ecosystem services^27^.

We normalized every indicator by considering the relative position of each value in the range between the real minimum (referred to as 0%) and real maximum values (100%) (‘min–max normalization’) to obtain a unitary performance index, *P*_i_. The minimum is considered to be the least and the maximum the most desirable value. Other approaches have used a hypothetical indicator value of zero as the minimum^28^,^29^, although zero is rarely included in the set of possible results. For example, as plants always store carbon, assigning a value of zero carbon to any kind of vegetation is not realistic. Moreover, our min–max normalization allows us to use indicators for which negative values are possible (for example, economic indicators). To combine indicators into a higher-ranked category—the key element index *P*_k_—we averaged the *P*_i_ values. We then formed the ecological and socio-economic index value of each land-use option, *WP*_o_, by calculating the average of its *P*_k_ indices. We applied the ‘more is better’ principle for most indicators. However, for ‘overland flow’ and ‘payback period’, the ‘less is better’ principle was used. This means that applying our scheme requires some local experience to form a meaningful assessment of each indicator.

Finally, we use sensitivity scenarios to test the robustness of our assessment approach. In one scenario, we account for the size of the differences by weighting the indicator values by their relative range of variation (objective weighting), because through our normalization, even small differences in indicator values are scaled between 0 and 100%. In another scenario, we test the impact of uncertainty by using both pessimistic and optimistic estimates (based on 95% confidence limits). After the pessimistic and optimistic indicators are normalized to create performance indices, their range is used to evaluate the robustness of our assessment system. The results of the uncertainty analyses are described in ‘Synopsis and sensitivity of indices’.

### Ecological indicators

Carbon relationships characterize the uptake and accumulation of carbon—a primary ecosystem function that is a pivotal part of provisioning (for example, fodder for cattle or timber), regulating (storage of atmospheric carbon) and life supporting (organic matter to improve soil quality) ecosystem services. We use three indicators for this assessment: biomass production, whole plant-cover carbon accumulation and soil organic carbon (Table 3 and Supplementary Tables 1 and 2). Carbon relationships for abandoned pastures assume equilibrium between production and death of bracken leaves and rhizomes^30^. For the tree plantations (*Alnus* or *Pinus*, see Supplementary Methods and Supplementary Tables 3 and 4), average values for biomass production over a 20-year period are computed, which account for losses from thinning and mortality.

Annual biomass production of bracken fern on abandoned pastures is the second highest among the options investigated. Thus, owing to the combination of the carbon present in this biomass and the organic carbon of the soil, abandoned areas rank intermediate in terms of total C-sequestration. The annual (20-year average) biomass production in the tree plantations is relatively low (see Supplementary Methods and Supplementary Fig. 1 for discussion). The corresponding average total carbon stocks *in planta* are slightly lower than those in the abandoned areas. However, when carbon in the accumulating litter layer is considered (Supplementary Table 3), total carbon sequestration in the tree plantations is in the same range as that calculated for abandoned pastures (Table 3). In both pasture types, *Setaria* is planted after bracken control. A nearly homogeneous grass canopy has been achieved after 1.5 years. In the ‘low-input’ variant, nutrient shortage strongly limits growth, even before weeds come up. After two rounds of (simulated) grazing, equilibrium biomass production is established with an above- to below-ground average ratio of 0.05. Owing to the very low C content in the standing above-ground biomass, C-sequestration potential in low-input pasture is the lowest of all the land-use options investigated. In contrast, fertilization of pastures in the high-input alternative results in an almost two-fold (over low-input pasture) and in some cases even a six-fold increase (over tree plantations) in above-ground biomass production. All of the grass leaf biomass except the basal 20 cm is removed in each of the three (simulated) grazing rounds.

The annual biomass production differs among the five ecosystem alternatives by a factor of 6.5 and plant-bound carbon by a factor of 2.6. The amount of carbon sequestered by each is similar (*Δ*~20%) due to the soil-bound fraction, which is high in all options. Because of high annual biomass production, the indices for ‘Carbon relationships’ are high for intense pasturing, moderate for both abandoned land and *Pinus* plantation, and low for low-input pasture and *Alnus* plantation.

Climate regulation is another important function of ecosystems, and the type and structure of the ecosystem directly influences the nature of surface–atmosphere exchanges. Thus, large-scale land-use changes elicit changes in both microclimate and the climate regulation function of an ecosystem^31^. The main drivers of this are changes in energy balance, surface roughness and evapotranspiration (ET), all of which link atmospheric to hydrological functions^32^. Here we calculate water and momentum fluxes (turbulence production, an important land–atmosphere feedback parameter) for a 20-year period using the coupled SoBraCo—catchment modelling framework (CMF)^33^ (see Methods and Supplementary Methods), to derive indicators for the intensity of surface–atmosphere exchanges.

The main components of microclimate, ET and turbulence production (M-flux, or the sum of zonal and meridional momentum fluxes) differ among the various land-use options (Table 4).

We find ET, the majority of which is plant transpiration, to be similar in the two types of tree plantation and significantly higher here than in the abandoned area. Periodic removal of biomass from the active pasture options leads to a decrease in transpiration, resulting in an overall ET lower than that in the tree plantations, but still higher than that of the abandoned pasture. Turbulence production is very high in tree plantations, whereas re-established pasture performs similarly to abandoned pasture.

Altogether, the tree plantations mimic the climate regulation function of a natural forest better than the options without trees. Afforestation with the broadleaf *Alnus* is even more effective in this regard than with *Pinus*.

Hydrological regulation performances of the various restoration options are crucial elements in assessing their potential for mitigating the adverse effects of water (such as erosion) but also in controlling the quantitative supply of water. We simulate below-ground water cycles using the well tested CMF^34^ (see Methods, Supplementary Methods and Supplementary Tables 5 and 6). Similar to the ambiguous effects that ecohydraulic processes can have on hydrological ecosystem services^25^, the investigated indicators might have a positive or a negative effect. For example, discharge (water volume) is a resource for hydropower^35^, and water that quickly leaves a system via overland flow and seepage prevents soils from becoming waterlogged. Therefore, rapid movement of water through the system can be considered positive. In contrast, seepage flow can also leach nutrients and overland flow can cause erosion, resulting in a negative effect from this indicator. To make our assessment more easily transferrable to sites with different objectives (high discharge or minimizing leaching/erosion), we calculate two separate indicators. The first indicator (*P*_k1_) combines a positive effect (discharge) and a negative effect (overland flow), while the second (*P*_k2_) considers both factors to be negative (Table 5).

Annual interception is low for pasture but high for abandoned pasture and *Pinus*. Water returned to the atmosphere through ET dominates the water relations in the tree plantations, while discharge driven by groundwater recharge is the main hydrological component of both active and abandoned pasture. The amount of water infiltrating the soil is offset by plant transpiration, and thus its total share is lower in the tree plantations. The fraction of overland flow is dependent on steepness of slope, level of soil compaction and vegetation cover. Steepness of slope remains constant in the model, but the other two factors are allowed to vary. The results for overland flow range between 2 and 4% of precipitation, thus representing only a minor fraction.

If a large amount of discharge is desired (*P*_k1_), *Pinus* proves to be the best option, followed by abandoned pasture, *Alnus*, and the two active pasture options. In the second case (*P*_k2_), the ecological index value of the tree plantations increases considerably. Thus, ranking can also depend on location. On steep slopes with soils with high levels of conductivity water retention is more desirable, whereas on flatter ground with compacted soil discharge is more important.

Soil quality is essential in maintaining the long-term productivity, and thus the sustainability, of the provisioning services of our restoration options. The chosen indicators (Table 6 and Supplementary Table 7) are well known to vary in response to land-use change^36^, to support plant productivity^37^ and to contribute to soil biodiversity^38^.

The dominant soil types in the research area are Haplic or Folic Cambisols, and Mollic Cambic Umbrisols^36^. Burning the original forest fertilizes the mineral top soil, raises its pH and results in soils with higher contents of both organic carbon and total nitrogen, but extremely low phosphate availability^39^. The burnt litter layer slowly regenerates in tree plantations but not on pasture. Regarding the assessment of soil quality, we focus here on sustainable plant productivity^40^ (Table 6).

The soil quality determined for the various land-use options allows for a ranking, although two of the seven indicators—C- and N-mineralization rates—differ only moderately among the alternatives. Still, they are important here and in possible other applications of our assessment approach, as they are associated with different microbial communities^40^. Intense pasturing produces the best soils, with high organic carbon content, high microbial biomass and nitrogen mineralization rates, as well as high phosphate content. The relatively acidic pH in this variant results from artificial fertilization. The soil on abandoned pasture is inferior, and in its overall quality, similar to that under *Alnus* and low-input pasture. Afforestation with *Pinus* decreases the soil quality dramatically due to acidification and the concomitant decline in base saturation, soil organic and microbial carbon content. However, due to the acidic pH, availability of phosphate increases. As *Alnus* is able to fix nitrogen^41^, which improves most of the soil quality indicators, the soil under *Alnus* may get better with time.

### Socio-economic indicators

Economic investigations of the restoration options are imperative for analysing the likelihood that they will actually be implemented. Thus, we assess benefits from timber or food production based on their simulated market value (household data are given in Supplementary Data 1 and Supplementary Table 8). The analysis of the BAU land-use option (pasturing after forest clearing) provides data for comparison. We use the net present value (NPV, Supplementary Methods) to rank the benefits of each option from an economic perspective.

The NPV (calculated using a 5% discount rate) of the active land-use alternatives ranges from US$ 127 (low-input pasture) to US$ 1,435 ha^−1^ (*Alnus*), which is in accordance with results from previous studies^42^. Payback periods from 10 (intense pasture) to 18 years (low-input pasturing) are required to recoup initial investment costs (Table 7). Among the pasture options, the intense variant is best in economic terms, with an NPV of US$ 1,060 ha^−1^. To simulate a greater preference for immediate net revenues with low initial costs, an 8% discount rate is also tested. In this case, the relative position of leaving land abandoned improves, as the NPVs of the active management alternatives decrease under these conditions. Intense pasture (13 years) and afforestation (16 years), however, still break even within the time span considered.

The land-use variants differ widely in the distribution of net revenues over the 20-year time period. For both afforestation options, we find only 3 years with positive, albeit high, net revenues (Supplementary Table 9), while the pasture options generate positive net revenues in 18 years. Owing to the concentration of net revenues in only 3 years, the diversification of annual market and production risks in the afforestation options is not comparable to that of the pasture options, for which the ‘averaging effect’ is much stronger. The uncertainty of the intense pasture’s NPV is thus only around 50% of that of the tree plantations.

From a farmer’s perspective, all restoration options must be compared with the BAU option of land use in the study region^43^ (explained in Supplementary Methods). All restoration options tested are less favourable from an economic point of view than BAU, for which the NPV (US$ 1,435 and 1,765 ha^−1^ at 8% and 5% discount rates) is always higher than that of the best restoration option. The annualized differences between the NPVs (8% discount rate) of BAU and the restoration scenarios are US$ 87±52 ha^−1^ per year (*Alnus* as reference) or US$ 100±40 ha^−1^ per year (intense pasture as reference). These amounts suggest the order of magnitude of the financial transfers that might be required to convince farmers to establish one or more restoration options.

Social preference in the context of this method represents information beyond that contained in the economic indicators. In addition to the tangible values reported above, people tend to implicitly include intangible cultural values when expressing their preference for land-use options. Thus, the success of recommendations regarding land use depends largely on this indicator, as farmers must evaluate whether a particular option fits not only into their overall economic, but also into their household and socio-cultural situations^44^.

Using standardized questionnaires (Supplementary Methods), we asked Mestizo and Saraguro farmers about their preferences and find an inclination towards afforestation (Table 8 and Supplementary Table 10). Farmers of both ethnic groups list ‘lack of timber’ and ‘shortage of labour’ as the main reasons for this preference. Without the benefit of subsidies, farmers of both ethnic groups prefer *Alnus* over *Pinus*; however, given the possibility of external subsidies, the Mestizos show a slight preference for *Pinus*. With respect to repasturization, low-input pasture appears to be more attractive than the intense option. Concerns about adverse ecological effects and the costs for fertilizer are the main reasons given by Mestizos for preferring low-input pasturing: more than one fourth of Mestizos interviewed (10 out of 37) believe that agrochemicals damage or ‘sterilize’ the soil. The Saraguros, however, state a higher preference for intense pasture if subsidies are available, but nevertheless consider reforestation with *Alnus* to be the best option.

The interviews show a clear preference for tree plantations. Interestingly, leaving areas abandoned is not favoured at all. Farmers express willingness to re-utilize abandoned areas but are less ready to invest high upfront costs or substantial labour to do so. Differences in the acceptance level even among different ethnic groups support the necessity and usefulness of this indicator, especially when applied in other regions.

### Synopsis and sensitivity of indices

**Figure 1:**
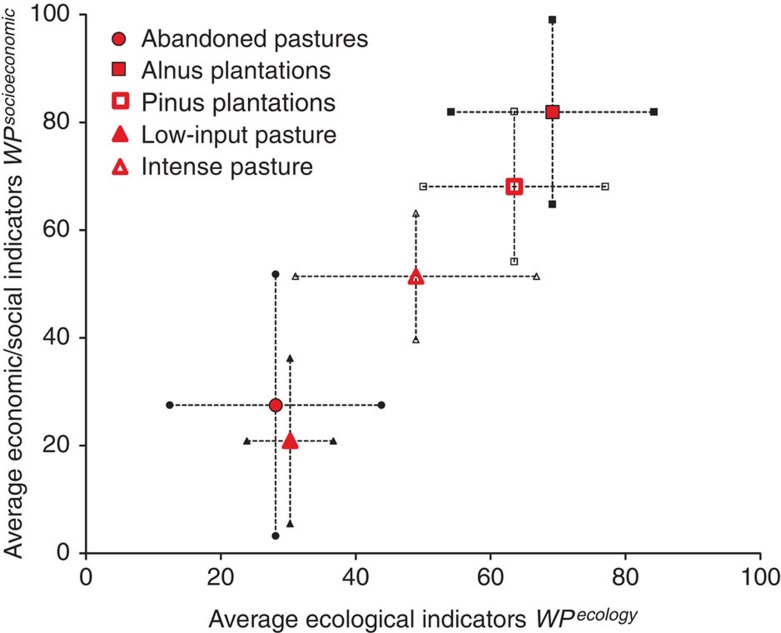
Ecological versus socio-economic index values. Average of key element—*P*_k_—indices of the five investigated options of land use if water retention is considered positive. Error bars (whiskers) indicate±s.e.m., coefficient of correlation is *ρ*=0.99 (*t*_ρ_=11.77; *p*_*t*_<0.001); the statistic of a Kruskal–Wallis one-way analysis of variance is *H*=13.4 (*p*_H_<0.01) for differences between overall average index values (*n*=8 key elements for each land-use option). *A priori* hypotheses about differences between single land-use options or groups of land-use options are tested as statistical contrasts using rank transformed data with: Ab, abandoned pastures; A, *Alnus*; P, *Pinus*; L, low-input pastures; I, intense pastures. Contrast 1, associated with the hypothesis (A+P+L+I)/4>Ab, tests if restoration options on average improve ecological and socio-economic values, and results in a significant *t*_c1_*=*2.3 (*p*_c1_<0.025). Contrast 2, associated with the hypothesis (A+P)/2>(I+L)/2, tests if afforestations perform better than pasture, and results in a significant *t*_c2_*=*3.1 (*p*_c2_<0.025). Contrast 3 focuses on the hypothesis A>P and tests if *Alnus* outperforms *Pinus*, and results in a nonsignificant *t*_c3_*=*0.9. Contrast 4, associated with the hypothesis I>L, tests if intense pastures perform better than low-input pastures, and results in a weakly significant *t*_c4_*=*1.6 (*p*_c4_<0.100) (Supplementary Table 22).

To gain more insight into the possible trade-offs between ecological and socio-economic ecosystem indices, and to support science-based decision making, we analyse the correlation between the average ecological and socio-economic key elements (Fig. 1).

**Figure 2:**
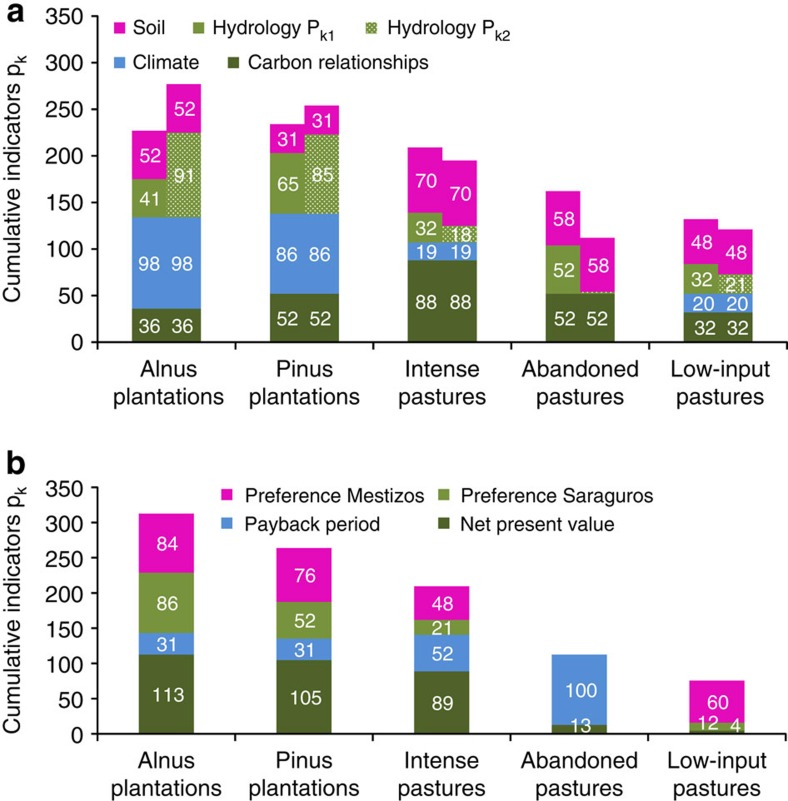
Accumulated index values. (**a**) Summed index values on ecological indicators are shown for the five land-use options with *P*_k1_: discharge considered positive (left columns) and *P*_k2_: water retention preferred (right columns). (**b**) Summed index values on socio-economic indicators for the five land-use options are depicted. Preferences distinguish between indigenous Saraguro and Mestizo settlers. With payback periods, we measure how long settlers will need to receive their money invested back and the NPV is the sum of all appropriately discounted net revenues.

Spanning considerable ranges, the ecological and socio-economic indicators are strongly positively correlated and show a low degree of trade-off—+0.99 with high water retention, and +0.94 with high discharge considered most desirable. Leaving areas abandoned and low-input pasture both appear less efficient than the other options. Ranking by the ecological indices alone places afforestation on top, irrespective of the hydrological key element used (Fig. 2a).

*Alnus* plantations rank slightly higher than *Pinus* plantations, followed by intense pasture. Afforestation and intense pasture both rank higher than the original state of ‘abandoned pasture’. Low-input pasture is ecologically equal to abandoned pasture when water retention is assessed as positive, but falls short when water discharge is more desirable.

The economic results (Fig. 2b) are supported by the analysis of the preferences obtained from the household survey, which show an affinity for afforestation among all respondents.

Sensitivity analysis (Supplementary Tables 11–21) shows our ranking to be sufficiently robust in this context and provide an indication of how this ranking might be affected by subjective weighting of key elements (Supplementary Fig. 2). The ranking remains the same when we account for the size of the differences by applying equation (3) (see Methods) in an attempt to prevent overestimation of small differences (Supplementary Fig. 2a). However, with this type of weighting, the ecological indicators distinguish less clearly among the restoration options. Although some ecological differences may be considered small (for example in N-mineralization), their importance may be high. Such differences are scaled appropriately with our approach. Another advantage is its relatively high immunity against uncertainty. By calculating the relative position of the indicator values in the range between their maximum and minimum, we obtain robust rankings that are largely unaffected by indicator uncertainty (see below). When using the 95% confidence limits to represent pessimistic and optimistic indicator estimates and account for uncertainties (according to equation (4) in Methods), some land-use options change their rank position, but only for single indicators. One example of this is the option pair *Alnus* and intense pasture, which trade positions between ranks 1 and 3 for the indicator NPV (8%) depending on whether the analysis is based on optimistic or pessimistic assumptions (Supplementary Table 20). However, where process-based model results contribute to the assessment, the individual (*P*_i_) and integrated (*P*_k_) indicator assessment scheme are very robust (Supplementary Tables 15–18). In sum, we do not see any significant overall variation in the average normalized indicators (Supplementary Fig. 2b,c). Only under pessimistic assumptions does the correlation between socio-economic and ecological indicators, and significance levels weaken (Supplementary Fig. 2c and Supplementary Table 22). Given the generally high level of robustness of our ranking system, we conclude that both our normalization procedure and assessment approach are reliable.

## Discussion

Assessing the potential for the provision of ecosystem functions and benefits from various restoration options using normalized ecological and socio-economic indicators is a novel approach in science-directed decision support. It is clear that any such assessment is context specific and dependent on the particular objectives of the decision makers in a given ecological and socio-economic context. Nevertheless, our study shows that the often ignored socio-economic indicators are essential components of a comprehensive assessment approach to guide the way to a possible implementation of desirable land-use options. Their extremely strong correlation with ecological indicators does, however, not necessarily mean that a cause–response relationship exists. It rather indicates that trade-offs between average indicators are low. Thus, to avoid assessing the consequences of restoration options overly optimistic, it is important to quantify the short-term socio-economic trade-offs when compared with BAU. Given this premises and combined with a thorough analysis of uncertainties, both the indicator system and the normalization procedure developed in our study are useful for comprehensive evaluations of ecosystem functions and benefits in other study regions.

The choice and quality of indicators are crucial issues. For example, our quantitative hydrological indicators showed realistic results when compared with other studies^45^,^46^. However, water quality could also be an important indicator^25^, as intense pasture requires the use of herbicides and inorganic fertilizer, some of which could end up in rivers and groundwater. This problem can be mitigated by proper handling, that is, application of any agrochemical only under suitable weather conditions. As overland flow is generally low (2–4% of precipitation), the volume of water for direct downhill transport of the chemicals is also small, reducing the risk of displacement. In fact, our observations of the control plots located down slope from herbicide-treated plots did not show any herbicide effect. Carryover effects of any of the herbicides applied via the soil to the subsequent pasture were also not observed^24^. A similar consideration applies to fertilizer treatment, as the poor soils act as strong nutrient sinks. Nevertheless, some leaching of nitrate cannot be completely ruled out, although we did not observe a statistically significant fertilization effect in reference plots situated down slope.

In contrast to other authors^28^, we did not use biodiversity as an indicator in our study, as it is very low in the options investigated and—even on long-abandoned pasture—is not at all comparable to that of the pristine forest. Stable shrubby vegetation made up of bracken and several prolific roadside species formed a closed canopy^21^, which can persist for decades (Supplementary Methods). Species richness of selected other groups of organisms such as birds and moths is also low compared with that found in pristine forests. Thus, the anthropogenic landscapes flanking forests are merely sinks for such species, mainly due to a shortage of food resources, nesting sites and other ecological factors necessary to provide suitable habitats. Consequently, the land-use options considered here represent ‘novel ecosystems’ that are expected to persist, rather than merely being an interim stage in the process of returning to a near-natural forest^47^,^48^,^49^. Natural grassland suitable for use as pasture does not occur in the research area and thus introduced grass species (*Setaria sphacelata* and *Melinis minutiflora*) are used. Some accompanying herbs, grasses and shrubs are indigenous, but most are cosmopolitans^50^, resulting in limited phytodiversity. Every hectare of natural vegetation—dense forest up to 2,800 m asl and shrub páramo above the tree line^51^—that is not cleared for production of short-lived pastures preserves much more of the biological diversity than pasture, afforested areas or abandoned lands can maintain.

Still, some barriers must be overcome to implement the advantageous restoration options, as all of them impose short-term economic trade-offs. The quantified trade-off of US$ 87 in opportunity costs resulting from afforestation with *Alnus*, or the US$ 100 ha^−1^ per year costs incurred when intense pasture is implemented are, however, subject to a high level of uncertainty. If we use the upper 95% confidence limit of the estimated costs to include the possible compensation amounts demanded with a 0.975 probability, we end up with approximately US$ 180 ha^−1^ per year to be transferred to farmers. Spending this money could be worthwhile, given the amount of CO_2_ emissions^42^ and losses of biodiversity, which could be avoided, and the other ecological benefits, which could be achieved if farmers were to re-utilize their abandoned lands rather than clearing natural forest. In our study area, the preservation of natural forests may prevent the emission of 272 Mg CO_2_ per hectare^43^. Consequently, a moderate price for CO_2_ emission allowances of US$ 7.5 per Mg would result in a NPV of US$ 2,040 ha^−1^, which is equivalent to an annualized payment of US$ 208 over a 20-year period (based on an 8% discount rate). Carbon markets could, thus, possibly cover the compensation amounts needed to convince famers to choose restoration options.

However, to improve conservation efficiency, transfers to landowners as rewards for conserving their forests—for example, under the REDD+ mechanism^52^ or other national programmes such as the Ecuadorian ‘Socio Bosque’^53^—should be made conditional on the implementation of restoration activities on abandoned land, considering also agricultural options in the future^54^. In regions with chaotic property rights regimes, as in our study area^44^, the implementation of the restoration options could also be supported by offering property rights contracts (possibly coupled with additional financial compensation). The size of the abandoned area, its accessibility and distance to the farm must also be considered in recommendations, as the advantages of afforestation increase with distance to farm, whereas those of intense pasture increase with increasing accessibility.

As a general conclusion, it appears important for farmers to receive appropriate education and financial support to highlight and strengthen the link between more long-term economic thinking and ecological considerations. Our study shows that preference analyses are crucial parts of studies on ecosystem functions and benefits. The preference expressed by the majority of subsistence farmers for restoring abandoned pasture areas through afforestation demonstrates that implementation of this option is realistic. Farmers could benefit from more moderate upfront costs, the lack of a need for further inputs of labour until thinning and harvest activities take place and flexibility with respect to the timber market, which ultimately results in a reduction in risk^54^. *Pinus* could be used as a nurse-tree species to facilitate regeneration of useful native trees. Restoration of abandoned pasture for intensive re-use may be more attractive on medium to large farms (50–100 ha), which are already integrated into agricultural markets and can afford higher upfront investments. The implementation of intense pasturing will require a higher level of input from consulting experts. Similar conclusions will be valid for other tropical mountain regions, from 1,500 m altitude up to the tree line.

As evidenced by the short-term economic trade-offs inherent to each of the restoration options, a farmer’s decision to afforest, re-cultivate pasture or leave areas abandoned depends—in addition to available labour capacity—on the availability of affordable financial support from government programmes or credit institutions. Studies such as ours can help raise awareness about possibilities for recultivating abandoned land, thus enhancing the effectiveness of incentive programmes, which could ultimately relieve pressure on natural ecosystems^42^.

## Methods

### Land-use options and period considered

We investigate two variants each of afforestation and pasture restoration as feasible options with respect to their ecological value, their economic benefits and the preferences for each option among both Mestizo and indigenous Saraguro farmers. A former pasture area that has been abandoned for 14 years is our reference. We consider a 20-year period to be a meaningful time span for the study, as it represents the common rotation time for tree plantations in the region.

### Data

The present work synthesizes the findings of a multi-disciplinary research initiative that started ecosystem studies in southern Ecuador in 1998. Since then, a solid knowledge base has accumulated with data that is used in this study. Models parameterized with field data are used to obtain the results for some indicators over the 20-year period during which we assume nearly constant environmental conditions. Other indicators were either measured directly in the field or obtained from interviews. Field data for carbon relationships and soil quality has been obtained from previous peer-reviewed work carried out by members of the multi-disciplinary research team^24^,^30^,^31^,^36^,^40^,^55^,^56^,^57^. Model-based indicators have been estimated with models published in peer-reviewed journals, which have been developed for or adapted to the study region. Model estimates include climatic^32^,^36^,^55^, hydrological^34^,^46^,^58^ and economic^42^,^43^,^59^ approaches. Only occasionally have models from other literature been used to complement our data^60^,^61^. The results of the interviews (Supplementary Table 10) to obtain data on the social preferences have not been published in peer-reviewed journals before. Details, as well as an assessment of the methods are presented in Supplementary Methods.

### Research area

The research area^62^ is located in the eastern range of the tropical Andes of southern Ecuador (3°58′30′′ S and 79°4′25′′ W). Our experimental sites were established on areas with a 35° slope located between 1,800 and 2,100 m asl, and covering a total area of abandoned pasture of 150 ha. Analysis of aerial photographs shows that forest clearing has been occurring since the 1960s, and pasture farming has been done for about the last 35 years. Because of heavy infestation by weeds—mostly bracken fern—many pastures were abandoned about 15 years ago.

### Normalization of indicators and statistical analyses

Unitary performance indices are calculated for each indicator (*P*_i_) and for each of the key elements (*P*_k_). *P*_i_ (equation (1)) reflects the relative position of a land-use option in the achievable range. *R*_i_ is the indicator value, i the land-use option, *R*_min_ the least desirable and *R*_max_ is the most desirable value for the indicator.


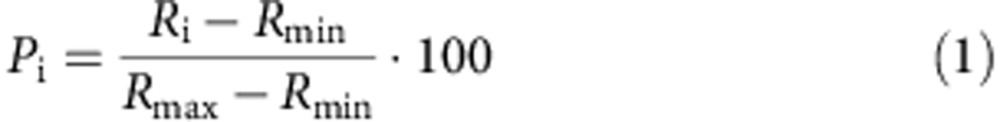


As equation (1) might result in inflation of small differences through its normalization approach, we impose an objective weighting factor, *w*_d_, proportional to the maximum achievable difference to test the robustness of our *P*_i_. Specifically, we use the total range of variation divided by the indicator’s maximum as the weight, *w*_d_, to account for the relative size of the maximum achievable difference and to see how our results change through this type of weighting (equation (2)).


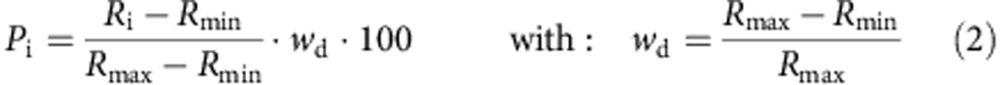


Adjusted according to equation (2), equation (1) then simplifies to equation (3):


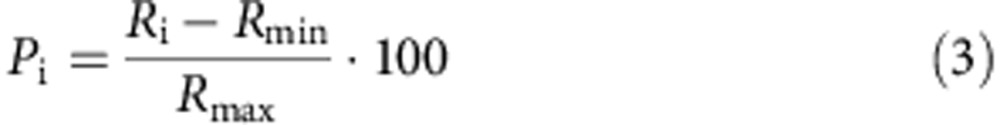


Although equation (3) constitutes a weighting of the normalized indicators, we also conduct sensitivity studies in which we test scenarios using either pessimistic or optimistic estimates for our indicators to identify possible impacts of uncertainty. To obtain the pessimistic and optimistic estimates, 95% confidence limits for the estimated indicators are used. The interpretation of a confidence limit as pessimistic or optimistic depends on what is desirable. If a high indicator value is desired (for example NPV), the lower confidence limit is considered to be the pessimistic (near worst-case) estimate. If instead a low indicator value is preferable (for example, payback period), the upper confidence limit is considered pessimistic (equation (4)).


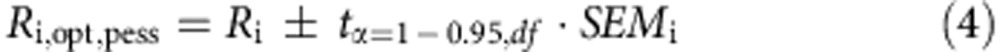



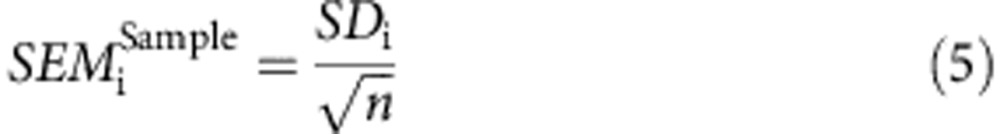



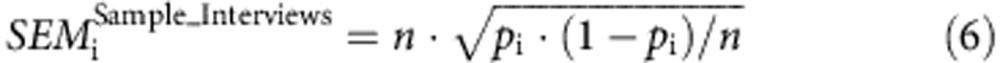



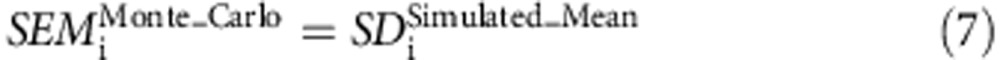


Here, *SEM*_i_ is the uncertainty associated with the estimated mean indicator value for restoration option i, commonly understood as the s.d. of the mean and *t*_*α=*1−0.95,*df*_ is a number obtained from a Student’s *t*-distribution, which is used to form a 95% confidence limit depending on the degrees of freedom, *df*. For indicator values derived from sampling, we obtain *SEM*_i_ by dividing *SD*_i_ (the s.d. among individual samples) by the square root of *n* (number of samples) (equation 5). For the interview data, the *SEM*_i_^Sample_Interviews^ (equation (6)) is the s.e. of the number of answers where a restoration option is chosen as the best or second best alternative. Here, *n* is the sum of all responses of ‘best’ or ‘second best’, and *p* is the relative frequency of the responses ‘best’ and ‘second best’ for that restoration option. In detail, *p* is the number of ‘bests’ and ‘second bests’ for option *i* divided by *n*—the sum of all answers for a given indicator naming these categories. For the model estimates, *SEM*_i_^Monte_Carlo^ is computed directly as the standard deviation, *SD*_i_^Simulated_Mean^, of the mean values derived from the simulated repetitions (equation 7). Finally, normalization of either *R*_opt_ or *R*_pess_ is carried out according to equation (1).

*P*_k_ (equation 8) is the average of all (weighted or not weighted) *P*_i_ values, which contribute to a particular key element (*n*_i_ is the respective number of indicators).


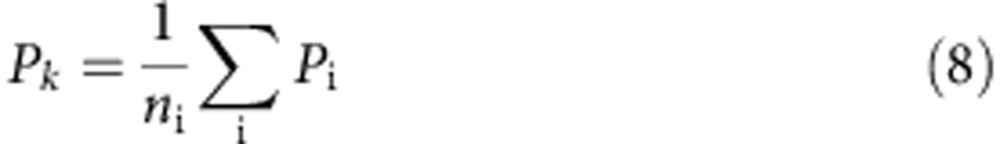


The ecological and socio-economic average of index values for each of the land-use options are determined by average performance indices (equation 9) (weighted or not weighted), where *o* is the land-use option, *c* the category, *n*_k_ is the number of key elements and *w*_sub_ is a subjective weighting factor.


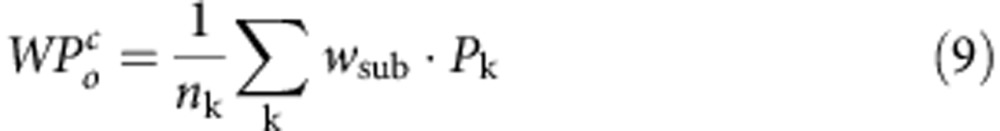


*w*_sub_ is set equal to 1 for standard analyses. For specific scenarios, however, we test subjective weighting factors to favour preferred key elements.

We compute Pearson correlations between ecological and socio-economic indicators for *P*_k_ values and associated *t*- and *p*-values (Fig. 1 and Supplementary Fig. 2). Given our—through normalization—truncated distributions of *P*_i_ index values, non-parametric Kruskal–Wallis analyses of variance served to test the impact of land-use options on the average *P*_k_ (*n*_k_=8 for each option). Associated with a one-way analysis of variance for rank-transformed data, contrasts based on *a priori* formulated hypotheses are computed finally to distinguish between the land-use options investigated (Supplementary Table 22).

### Biomass production and carbon content

On the abandoned areas (control plots), above-ground plant biomass (bracken leaves) was harvested individually from four 25-m^2^ plots and dried for further analysis, while below-ground biomass was estimated from roots and rhizomes extracted from soil cores (40 cm deep, 6 cm diameter, *n*=3 per plot). Aliquots of the dried plant material were analysed in a CNS-Analyser (vario EL III/elementar, Heraeus). Production of above-ground biomass was determined based on the amount of standing biomass and the life span of the bracken leaves^30^. Below-ground biomass production was estimated using the SoBraCo-model^33^. On the pastures, biomass production and total biomass were determined using 4 × 4 m plots with four repetitions per option during the second year of pasture management after complete removal of the harvest from the first year. Grazing was simulated by cutting the grasses and leaving a basal layer of 20 cm. At the end of the year, the grass was completely harvested. For calculation of the standing crop, see Supplementary Methods. Below-ground biomass was determined and root biomass production was estimated as described above, with cores taken below, near and between grass tufts. SEM was calculated according to equation (5). Based on data from the experimental plots as described in Günter *et al*.^63^, growth of the *Pinus* and *Alnus* trees over 20 years was calculated. This was done using regression curves to correlate dbh (diameter at breast height) and height with the independent variables age and tree density. Tree density is based on an initial density of 1,111 trees per hectare and an observed annual mortality rate of 2%. To establish the regression curves, *Pinus* was recorded on two sites with 16 plots each (32 plots of 10.8 × 10.8 m, all 6 years old) and on one site with two circular plots (radius 20 m, 1,256 m^2^, 25 years old). Data for *Alnus* was measured on two sites, one with 14 and the other with 16 plots (30 plots 10.8 × 10.8 m, 7 years old) and one site with 10 plots (size on average 777 m^2^, 8 years old). Above-ground biomass and carbon content are estimated using both allometric equations and information adopted from the literature (Supplementary Methods). Uncertainty is modelled by means of Monte-Carlo (MC) simulation (3,000 repetitions for each afforestation option), where the coefficients of regression models are considered random. Regression coefficients as means, and their uncertainties, in the form of their s.e. allow us to draw randomly fluctuating coefficients for each simulation run to predict forest growth. The calculation of SEM refers to equation (7).

### Climate

The Soil-Vegetation-Atmosphere Transfer model and the vegetation growth model SoBraCo^33^ are used to assess changes in the climate regulation function of the land-use options. SoBraCo is a derivative of the properly validated community land model (CLM, see Lawrence *et al*.^64^ and Bonan *et al*.^65^). The main difference between SoBraCo and CLM is that in SoBraCo, the calculations used in CLM for some atmospheric variables are replaced by direct forcing with observational data from a specifically designed micro-meteorological station for an average reference year (2008; refer to Supplementary Methods). Hourly environmental forcing data for the 20-year modelling period is generated using the reference period for the forcing variables (solar irradiation, air temperature, relative humidity, wind speed, rainfall, soil water content and soil temperature) and continually re-applying the annual data set over the entire period. Required plant-specific model parameters are derived from measurements at both leaf and root levels at the study site and from data available from the literature (for more details, refer to Supplementary Methods and Supplementary Table 12). CMF is directly coupled with the SoBraCo-model using a Python interface^58^.

Time series of leaf area indices (LAIs) and vegetation height used for parameter forcing for the coupled model over the 20-year period are calculated using field measurements (Supplementary Fig. 1). For *Pinus*, LAI is estimated based on dbh^60^. For *Alnus*, observed leaf numbers from experimental plots and mean leaf area^61^ are used to estimate the mean allometric leaf area (*LA*_allom_) per tree, as *LA*_allom_*=*EXP(−0.22+1.297 × ln(dbh)). *LA*_allom_ and the actual number of trees is used to scale LAI up to the plantation level. LAI for grass and bracken are directly derived from LAI field measurements^56^. Uncertainty analysis (for climate and hydrology) of the coupled SoBraCo-CMF model framework is conducted for forcing variable and parameter uncertainties of climate and hydrological indicators, the latter with the help of more than 3,000 MC simulation runs and subsequent calculation of the SEM using equation (7). Based on sensitivity studies and a literature survey, eight (SoBraCo)+two (CMF) model parameters shown to have the biggest influence on model output are chosen (see Supplementary Methods). Most parameters and the form of their probability density functions are based on field observations (for parameters and sources, see Supplementary Tables 12 and 13). The robustness of the integral rating scheme (*P*_k_) regarding climate and hydrological indicators is tested by comparing the range of the *P*_i_ grading considering forcing variable uncertainty (Supplementary Tables 17 and 18). A scenario using 95% limits for the indicator values (following from equation (4)) derived from the MC analysis (parameter uncertainty) has also been tested (Supplementary Tables 15 and 16).

### Hydrology

The CMF^34^ is used to simulate hydrological processes in a one-dimensional soil column and to quantify water fluxes. This model effectively meets the challenges and provides the opportunities called for in hydrological models to support decision making outlined by Guswa *et al*.^66^ Similar to the finite volume method used by Qu and Duffy^67^, CMF discretises the soil column into soil layers serving as water storages. We use the Richards equation to simulate water flux between cells. Eight soil layers of increasing thickness from the top downwards, each with unique hydraulic properties, are summed to reach a total column depth of 1 m. Water leaving the soil column is routed to the ground water using a Dirichlet boundary condition with a constant negative pressure. An average slope similar to the slope occurring at the site is used. The CMF water balance is as follows: Δstorage=rainfall—ET—overland flow—ground water seepage. Groundwater seepage plus overland flow is summed to derive the area-specific discharge. Saturated hydraulic conductivity (*k*_sat_) for the scenarios is adapted in accordance with local measurements for pasture and forested sites presented by Huwe *et al*.^57^ To account for the highly conductive litter layer and roots that create additional pore space, the *k*_sat_ values of the three top soil layers are increased. ET and throughfall are calculated using the SoBraCo-model^33^, which shows realistic results. For example, the rate of throughfall forwarded to the hydrological model by the plant growth model SoBraCo is well within the range found in other studies in adjacent tropical mountainous rainforest sites^45^ (between 88% and 97% of precipitation). Saturated soil depth is set to −2 m below soil surface to initialize the model and provide uniform initial conditions. To emphasize the impact of vegetation type, uniform parameters for soil are used for all of the land-use options (Supplementary Methods). Overland flow is simulated as saturation excess. For the analysis of uncertainty, refer to the key element ‘Climate’.

### Soils

The quantification of our ecosystem function is based solely on empirical data measured on five plots in 2011. Soil samples were taken with an auger (diameter: 6 cm). A pooled soil sample of six replicates per plot was analysed for the 0–5 and 5–10 cm depth intervals. Soil organic carbon and total nitrogen (N) were quantified using a CNS-Analyzer (vario EL III/elementar, Heraeus). Fresh samples were used for all other measurements, but the data were calculated for dry weight (105 °C). Plant available PO_4_-P was determined by the Bray-P method^68^ and the chloroform-fumigation extraction method was used^69^ for microbial carbon. Gross N mineralization was measured by the ^15^N-isotope pool dilution method and soil organic carbon mineralization by CO_2_ evolution during 14 days of incubation^70^. Soil pH was determined in deionized water. Soil data were gathered for restored pasture beginning 2 years after re-establishment and for tree plantations in the oldest existing plots. Calculation of SEM refers to equation (5).

### Economics

The modelled (afforestation) or measured (pasture) biomass production in the form of either timber volume (afforestation) or fodder for a specific number of cattle (pasture) forms the ecological data to be evaluated using local prices and costs. Local historical timber prices and harvesting expenses, reported in Supplementary Data 1, were applied to the afforestation areas to estimate the net revenues from timber production (see Supplementary Table 4 for biophysical timber production and 18 for financial household data). For pasture, extrapolation of simulated grazing generates expected fodder yield, and the number of cattle that can be fed is computed based on the measured nutrient value of the grass. Milk and meat yield, as well as corresponding prices and costs are given in Supplementary Data 1. The distribution of net revenues over the 20-year time period forms the basis for economic valuation (Supplementary Table 9). The sum of all discounted net revenues (NPV) is used to evaluate the economic returns from the various land-use options and discounting based on discount rates of 5 or 8%. Payback periods are calculated based on discounted net revenues (Supplementary Methods). For the scenario ‘pasturing after forest clearing’ (BAU), upfront net returns from forest clearing and pasture establishment were obtained from Knoke *et al*.^43^ and combined with subsequent net revenues from low-input pasture management (Supplementary Table 9). Uncertainty is modelled by means of MC simulation (3,000 repetitions for each restoration option) for the annual net revenues used to calculate NPV and payback periods. Here, net revenues are drawn as random variables for every single year of the 20-year period considered from a normal distribution with the previously estimated expected net revenue as the mean and the s.d. indicated in Supplementary Table 9. After completing the annual simulation of net revenues over the entire 20-year period, a random NPV and payback period are computed for all iterations and SEM is calculated according to equation (7). Year-to-year correlation is set to zero. This appears reasonable, because average year-to-year correlation of revenues (average price obtained times quantity produced) for 10 South American countries was 0.04±0.21 according to a data set used by Knoke *et al*.^59^ Uncertainty coefficients for the land-use options in the study area are derived from coefficients of variation published in an earlier analysis^43^ in our study area. The coefficients of variation reflect compounded s.d. of prices and productivities from Food and Agriculture Organization of United Nations time series data, as well as s.d. caused by failure due to fire (coefficients in Supplementary Table 9).

### Social assessment

The preferences of Saraguro and Mestizo farmers for the five proposed land-use options were determined using a standardized questionnaire (Supplementary Methods). This includes questions regarding the land-use preferences of the farmers and their arguments for preferring particular land-use options, as well as information about household composition, ownership of abandoned land and reasons for its abandonment. Two scenarios are tested—one in which farmers reclaim the abandoned areas using their own means (without subsidies) and a second in which farmers receive financial support for major inputs from external agencies (with subsidies).

## Author contributions

T.K., E.B., J.B., P.P., L.B., R.M., M.W., B.S., S.G. and N.A. designed research and wrote the paper; P.H., U.H., A.G., M.L.S., K.R., A.T., B.S., B.C., L.M.C., D.W., X.P. and J.M. analysed data and performed research.

## Additional information

**How to cite this article:** Knoke, T. *et al*. Afforestation or intense pasturing improve the ecological and economic value of abandoned tropical farmlands. *Nat. Commun.* 5:5612 doi: 10.1038/ncomms6612 (2014).

## Supplementary Material

Supplementary Information Supplementary Figures 1-2, Supplementary Tables 1-22, Supplementary Methods and Supplementary References

Supplementary Dataset 1 Household data, harvesting expenses, and milk and meat production costs.

## Figures and Tables

**Table 1:** Characterization of the land-use options investigated.

**Land-use option**	**Land preparation**	**Establishment**	**Management**
Abandoned pastures: leaving areas abandoned	None	None	None
*Alnus*: afforestation with native *Alnus acuminataPinus*: afforestation with exotic *Pinus patula*	Initial removal of weeds (bracken)	1,111 Trees per hectare	Weed control in years 1 and 2, 2 thinning campaigns (years 12 and 16)
Low-input pastures: repasturization with low-input management after mechanical weed control	1 Year with 4 recurrent cuttings of bracken	32,400 Grass plantlets per hectare	1 Weed control/year2 Grazing rounds/year
Intense pastures: repasturization with intense management after chemical weed control	9 Months with 3 recurrent herbicide applications	As above	3 Grazing rounds/year3 Fertilization campaigns/year

**Table 2:** Categories, key elements and associated indicators, data sources.

**Categories**	**Key elements**	**Indicators**	**Data source**
Ecological functions	Carbon relationships	Biomass production, carbon *in planta*, soil organic carbon	Afforestation: statistical regression models, parameters estimated from field data; pastures and abandoned pastures: field data plus process-based model for annual below-ground biomass production
	Climate regulation	Evapotranspiration, momentum flux	Process-based models, most model parameters estimated from field data
	Hydrological regulation	Surface flow, groundwater recharge, area-specific discharge	
	Soil quality	pH, soil organic carbon, base saturation, carbon in microbial biomass, C-mineralization, N-mineralization, PO_4_-P	Field data
Socio-economic benefits	Net present valuePayback period	5% and 8% discount rates	Evaluation of timber and cattle products with market prices and costs (obtained from household surveys supplemented by data of the Food and Agriculture Organization of the United Nations)
	Saraguro preferenceMestizo preference for each land-use option	Saraguros asked with and without the option of subsidiesMestizos asked with and without the option of subsidies	Standardized questionnaires

**Table 3:** Rating of the key element ‘Carbon relationships’ (indicator value±s.e.m.)*.

**Land-use option**	**Annual biomass production**		**Carbon stocks**						**Carbon relationships**
	**(Mg ha**^−**1**^ **per year)**	***P***_**i**_	**Carbon** ***in planta***†		**Soil organic carbon**‡		**Total carbon**		***P***_**k**_
			**(Mg** **ha**^−**1**^**)**	***P***_**i**_	**(Mg** **ha**^−**1**^**)**	***P***_**i**_	**(Mg** **ha**^−**1**^**)**	***P***_**i**_	
Abandoned pastures	31.8±4.8	57	33.0±2.9	100	87.3±5.3	0	120.3±6.9	85	52
*Alnus*	7.7±0.6	0	24.5±2.3	58	91.7±6.8	49	116.2±7.2	63	36
*Pinus*	8.9±0.4	3	29.6±1.4	83	93.5±4.6	69	123.1±4.8	100	52
Low-input pastures	26.5±4.4	44	12.5±1.2	0	91.8±4.9	50	104.4±6.5	0	32
Intense pastures	50.0±2.3	100	25.8±3.4	65	96.3±5.1	100	122.2±5.5	95	88

^*^Estimates for tree plantations from statistical-based regression models parameterized with field data; for pastures, all data from field measurements except annual below-ground biomass production, which was estimated by the process-based model SoBraCo^33^, with parameters derived from field data.

^†^Averaged over a 20-year period.

^‡^Organic layer and mineral top soil (0–20 cm depth).

**Table 4:** Rating of the key element ‘Climate regulation’ (indicator value±s.e.m.)*.

**Land-use option**	**ET**		**MF**		***P***_**k**_
	**(mm)**	***P***_**i**_	**(kg** **m**^−**1**^ **s**^−**2**^**)**	***P***_**i**_	
Abandoned pastures	928±3.80	0	0.018±0.00028	0	0
*Alnus*	1,597±4.10	100	0.285±0.01560	97	99
*Pinus*	1,410±1.12	72	0.294±0.00038	100	86
Low-input pastures	1,186±5.81	39	0.023±0.00003	2	21
Intense pastures	1,167±5.10	36	0.026±0.00040	3	20

CMF, catchment modelling framework; ET, evapotranspiration; MF, momentum flux.

*ET and MF are simulated with the coupled SoBraCo-CMF model^33^. The model is forced with data of a micrometeorological station^33^. Optical and physiological as well as soil model parameters are derived from field observations presented in Bendix *et al*.^56^, Silva *et al*.^33^ and from literature (for more details, refer to Supplementary Table 12 and Table 2 in Silva *et al*.^33^).

**Table 5:** Rating of the key element ‘Hydrological regulation’ (indicator value±s.e.m.)*.

**Land-use option**	**Overland flow**		**Area-specific discharge**			***P***_**k1**_	***P***_**k2**_
	**(mm per year)**	***P***_**i**_	**(mm per year)**	***P***_**i(+)**_	***P***_**i(−)**_		
Abandoned pastures	75±3.74	4	927±6.90	100	0	52	2
*Alnus*	38±0.84	81	283±3.95	0	100	41	91
*Pinus*	29±1.48	100	471±2.7	29	71	65	86
Low-input pastures	75±2.81	3	677±6.97	61	39	32	22
Intense pastures	77±2.93	0	695±6.11	64	36	32	18

CMF, catchment modelling framework.

^*^Reported values are based on process-based model simulated data from the coupled CMF-SoBraCo setup adapted to the local land-use option and forced by local climate data (see Table 4).

**Table 6:** Rating of the key element ‘Soil quality’ (indicator value±s.e.m.; *P*_i_ in parentheses)*.

**Land-use option**	**pH**†	**SOC (%)**	**BS (%)**	**MBC (mg** **kg**^−**1**^**)**	**C-min (g CO**_**2**_**-C per kg SOC)**	**N-min**‡ **(mg N kg**^−**1**^**per day)**	**PO**_**4**_**-P (mg** **kg**^−**1**^**)**	***P***_**k**_
Abandoned pastures	4.5±0.09 (98)	9.5±0.18 (55)	11.5±2.64 (21)	1,088±51 (65)	3.9±0.18 (100)	2.3±0.27 (65)	0.5±0.09 (0)	58
*Alnus*	4.3±0.04 (89)	7.9±0.67 (22)	30.4±1.79 (100)	1,065±80 (63)	3.1±0.13 (0)	2.7±0.49 (85)	1.3±0.22 (15)	53
*Pinus*	3.6±0.13 (0)	6.8±0.76 (0)	6.4±1.21 (0)	576±75 (0)	3.7±0.49 (75)	1.9±0.31 (45)	5.8±1.21 (96)	31
Low-input pastures	4.5±0.18 (100)	10.6±0.58 (76)	16.9±1.30 (44)	1,065±102 (63)	3.5±0.31 (50)	1.0±0.22 (0)	0.6±0.13 (2)	48
Intense pastures	4.1±0.09 (78)	11.7±0.40 (100)	11.9±1.30 (23)	1,359±65 (100)	3.2±0.27 (13)	3.0±1.12 (100)	6.0±1.79 (100)	73

BS, base saturation; C-min, carbon mineralization; MBC, carbon in microbial biomass; N-min, Nitrogen mineralization; SOC, soil organic carbon.

^*^Field data, SOC, BS, MBC, C-min; *n*=5.

^†^*P*_i_ calculated as delog pH based on a higher precision than indicated in the Table to obtain a higher ecological significance than the commonly used pH shown in the Table.

^‡^N-min: data shown is only for 0–5 cm soil depth.

**Table 7:** Rating of the ‘Economic’ key elements (indicator value±s.e.m.)*.

**Land-use option**	**Net present value for discount rate:**					**Payback period for discount rate:**				
	**5% (US$ ha**^−**1**^**)**	***P***_**i**_	**8% (US$ ha**^−**1**^**)**	***P***_**i**_	***P***_**k**_	**5% (years)**	***P***_**i**_	**8% (years)**	***P***_**i**_	***P***_**k**_
Abandoned pastures	0±0	0	0±0	20	10	0±0	100	0±0	100	100
*Alnus*	1,435±649	100	619±394	100	100	16±3	11	16±4	50	30.5
*Pinus*	1,322±586	92	561±373	93	92.5	16±3	11	16±4	50	30.5
Low-input pastures	127±146	9	−156±129	0	4.5	18±6	0	32±4	0	0
Intense pastures	1,060±264	74	485±234	83	78.5	10±2	44	13±4	59	51.5

^*^Product (timber, milk and meat) quantities estimated based on tree and grass biomass predictions, and possible number of cattle calculated from simulated grazing rounds plus measured nutrition value of grass; local timber prices and harvesting costs, and prices and costs for milk and meat production contained in Supplementary Data 1; uncertainty from Monte-Carlo simulations, coefficients of variation^43^ from FAO time series data for prices and productivities, as well as from simulated fire risks based on remote sensing data.

**Table 8:** Rating of the key elements for ‘Social preference’; ‘answers’ refer to number of respondents who rate an option as best or second best (indicator value±s.e.m.)*.

**Preferred land-use option**	**Saraguros (22 interviews)**					**Mestizos (37 interviews)**				
	**Without subsidy**		**With subsidy**		***P***_**k**_	**Without subsidy**		**With subsidy**		***P***_**k**_
	**Answers**	***P***_**i**_	**Answers**	***P***_**i**_		**Answers**	***P***_**i**_	**Answers**	***P***_**i**_	
Abandoned pastures	4±1.9	0	0±0	0	0	5±2.1	0	0±0	0	0
*Alnus*	14±3.0	100	19±3.1	100	100	19±3.6	100	16±3.4	94	97
*Pinus*	12±2.9	80	9±2.6	47	63.5	15±3.4	71	17±3.5	100	86
Low-input pastures	5±2.1	10	3±1.7	16	13	12±3.1	50	14±3.2	82	66
Intense pastures	4±1.9	0	8±2.5	42	21	12±3.1	50	10±2.9	59	55

^*^Field data from standardized interviews.
