# Supplementary material for: Afforestation or intense pasturing improve the ecological and economic value of abandoned tropical farmlands
Source: Nat Commun. 2014 Nov 26;5:5612. doi: 10.1038/ncomms6612 (PMC4263169; doi:10.1038/ncomms6612)
Supplement: Supplementary Information — Supplementary Figures 1-2, Supplementary Tables 1-22, Supplementary Methods and Supplementary References [file ncomms6612-s1.pdf]

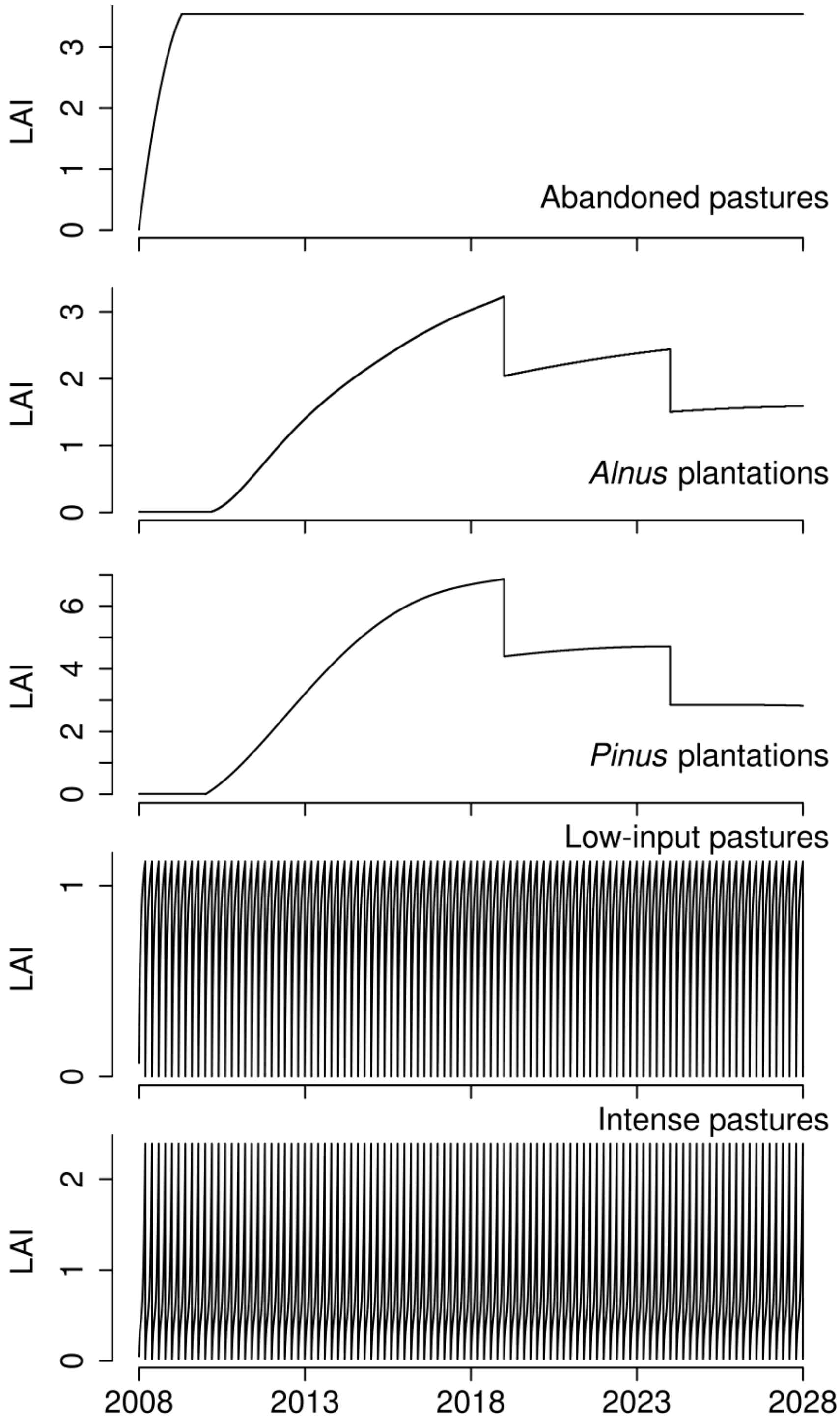

Supplementary Figure 1. LAI story line data for each land-use option used to force model parameters. Fluctuations in pasture and tree plantation scenarios represent grazing a timber extraction. LAI on abandoned pastures is kept constant over the 20-year period.

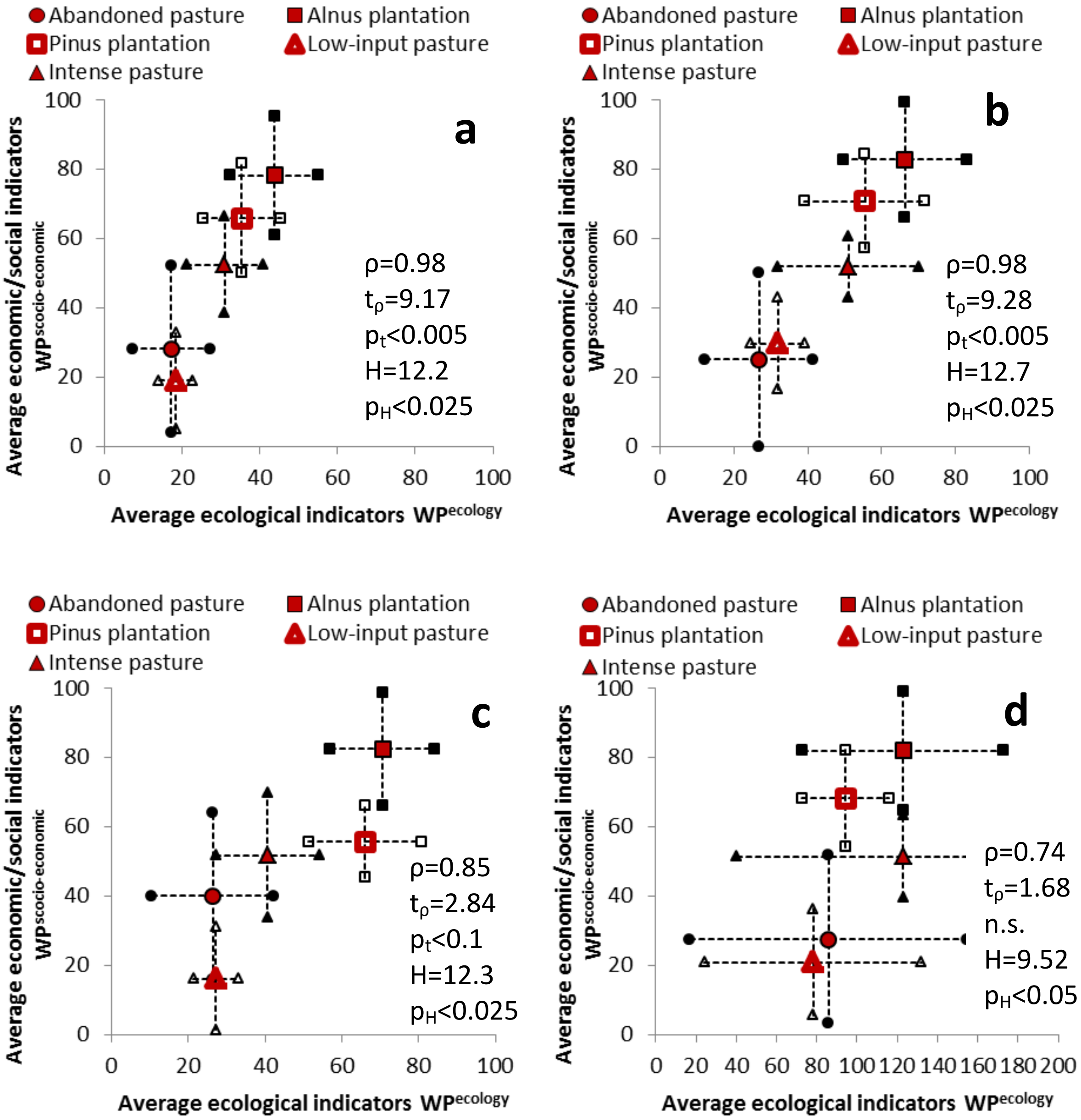

Supplementary Figure 2. Average of ecological and socio-economic index values (±SEM_{WP}) under various sensitivity scenarios. ρ is the coefficient of correlation between average index values with t statistic and p-value. H is the Kruskal-Wallis statistic for differences in overall index means of land-use options with p-value (n=8 key elements) **a.** Indicator values are weighted with their relative range of variation according to Eq. (3) in Materials and Methods (main text). **b.** Indicator values are estimated optimistically based on 95% confidence limits according to Eq. (4) **c.** Indicator values are computed pessimistically based on 95% confidence limits according to Eq. (4). **d.** Key element “Soil quality” is given a very high subjective weight, w_{sub}, of 5 instead of 1 to express a strong preference for food production, while all other key elements have a weight of 1, given Eq. (9). This puts intense pasture at first place for the ecological assessment. See Supplementary Table 22 for statistical contrasts between single land-use options.

Supplementary Table 1. Total yield (DM) and fodder quality of *Setaria* pastures after restoration and improvement through fertilisation.

Treatment	Biomass yield [kg ha^{-1} y^{-1}]	Content [g kg^{-1} DM]			
		Crude protein	P	Ca	N
**Restored pasture**					
Low-input pastures	1,240	38.2	0.69	2.21	6.12
Intense pastures	6,640	37.8	0.93	1.79	6.05
**Long standing pastures* with various fertilisers applied**					
Control	9,020	83.5	1.76	4.31	13.36
Urea	9,750	91.3	1.51	4.17	14.61
Rock phosphate	9,120	85.5	2.49	4.90	13.68
Urea + rock phosphate	11,190	79.3	2.08	4.61	12.68

*Data adopted from Potthast et al.[1]

Supplementary Table 2. Partitioning of biomass production (mean ± SEM) between above- and below-ground fractions for each of the five land-use options calculated on a per-year basis. Coefficient of variation for above ground biomass in parentheses.

Land-use option	Biomass production [Mg ha^{-1} y^{-1}]			
	Above ground	Above ground (with grazing)	Below ground*	Total**
Abandoned pastures	9.2 ± 1.4 (0.15)	-	22.6	31.8 ± 4.8
Alnus plantations	6.1	-	1.6	7.7 ± 0.6
Pinus plantations	7.1	-	1.8	8.9 ± 0.4
Low-input pastures	1.2 ± 0.2 (0.17)	0.8 ± 0.1	25.3	26.5 ± 4.4
Intense pastures	6.6 ± 0.3 (0.05)	5.2 ± 0.3	43.4	50.0 ± 2.3

*No SEM available for simulated values.

**To obtain SEM for the total biomass production, the measured coefficient of variation of above ground biomass production was used for the pasture options and for afforestation it was obtained through Monte-Carlo simulation (see Material and Methods main text).

Supplementary Table 3. Standing crop (mean ± SEM) and carbon stocks in each of the five land-use options calculated on a per-year basis. Where not otherwise mentioned: Data from field measurements.

**Land-use Option**	**Biomass average standing crop** [Mg ha^{-1}]			**C-content in roots and rhizomes** [%]		**Average carbon stock** [Mg/ha]					
	Above ground	Below ground	Total	Above ground	Below ground	Above ground	Root & Rhizome	Carbon in planta	Org. layer	SOC	Total
Abandoned pastures	6.1 ± 0.9	69.9 ± 6.7	76.0 ± 7.6	49[#]	43[#]	3.0 ± 0.4	30.0	33.0 ± 2.9	-	87.3 ±5.3	120.3
Alnus plantations*	40.7	8.3**	49.1	50	50	20.4	4.2	24.5 ± 2.4***	7.9 ± 0.3	83.8 ± 6.8	116.3
Pinus plantations*	49.1	10.1**	59.2	50[##]	50[##]	24.6	5.0	29.6 ± 1.5***	13.0 ± 1.1	80.5 ± 4.6	122.7
Low-input pastures	0.7 ± 0.1	29.9 ± 3.0	30.6 ± 3.1	44[#]	41[#]	0.3 ± 0.1	12.2	12.5 ± 1.2	-	91.8 ±4.9	104.4
Intense pastures	3.0 ± 0.1	59.9 ± 8.3	62.1 ± 8.4	44[#]	41[#]	1.3 ± 0.1	24.5	25.8 ± 3.4	-	96.3 ±5.1	122.8

*Averages computed over the entire production period (20 y).
**Calculated with a root:shoot ratio of 0.205 ± 0.036 (median, SEM) for tropical/subtropical moist forest/plantations according to Mokany et al.[2]
***SEM obtained through Monte-Carlo simulation (see Material and Methods main text).
[#]Analysed with a Vario EL III/elementar analyser (Heraeus)
[##]Acosta-Mireles et al.[3]

Supplementary Table 4. Stand characteristics for *Alnus acuminata* and *Pinus patula* obtained from a statistical based model (see material and Methods main text). Thinnings after 12 and 16 years with 40% of N_r removed each time; mortality rate = 2% of N_r.

							Standing crop							Removed crop		
	Age	N_r	N_d	Dbh [cm]	Ba [$m^2 ha^{-1}$]	h_t [m]	v_t [$m^3 ha^{-1}$]	v_c [$m^3 ha^{-1}$]	bm_a [Mg ha^{-1}]	bm_b [Mg ha^{-1}]	bm_t [Mg ha^{-1}]	C [Mg ha^{-1}]	LAI	N_{rm}	v_t	v_c
Alnus	0	1111	0	0.00	0.00	0.00	0.00	0.00	0.00	0.00	0.00	0.00	0.00	0	0.00	0.00
	4	1025	86	6.22	3.12	5.50	8.86	2.16	7.11	1.46	8.57	4.28	0.88	0	0.00	0.00
	8	945	166	14.88	16.45	9.64	81.29	28.97	45.85	9.40	55.25	27.62	2.52	0	0.00	0.00
	12	516	239	20.94	17.77	12.01	109.10	45.08	53.60	10.99	64.58	32.29	2.14	356	49.83	9.62
	16	282	279	25.55	14.45	13.65	100.60	45.32	45.60	9.35	54.95	27.47	1.51	194	49.74	10.61
	20	260	301	28.44	16.50	14.63	122.99	58.04	53.39	10.95	64.34	32.17	1.60	260	91.39	20.53
Pinus	0	1111	0	0.00	0.00	0.00	0.00	0.00	0.00	0.00	0.00	0.00	0.00	0	0.00	0.00
	4	1025	86	9.18	6.78	7.31	18.67	0.00	17.23	3.53	20.77	10.38	1.96	0	0.00	0.00
	8	945	166	16.30	19.73	10.82	81.71	12.11	55.68	11.41	67.10	33.55	5.99	0	0.00	0.00
	12	516	239	21.01	17.89	13.86	95.69	58.23	61.57	12.62	74.19	37.09	4.51	356	43.55	6.60
	16	282	279	24.52	13.30	16.33	84.26	63.72	53.03	10.87	63.90	31.95	2.85	194	41.09	12.39
	20	260	301	26.87	14.73	17.34	99.34	81.04	61.98	12.71	74.68	37.34	2.82	260	72.68	25.93

N_r = number of remaining trees; N_d = cumulative number of dead trees; Dbh = mean diameter at breast height Ba = basal area; h_t = mean total height; v_t= total volume without bark; v_c = commercial volume without bark; bm_a = above-ground biomass; bm_b = below-ground biomass; bm_t = total biomass; C = carbon sequestration; LAI = leaf area index; N_{rm} = number of trees removed.

Supplementary Table 5. Soil parameterisation and saturated hydraulic conductivity (k_{sat}) for CMF. Soil texture was parameterised identically for all land-use types.

Soil layer	Depth [cm]	Texture [%]			k_{sat} [m d^{-1}]	
		Sand	Clay	Silt	**Pasture scenarios**	**Afforestation scenarios**
1	0.0-1.5	28.8	31.9	39.3	20.0	40.0
2	1.5-4.0	28.8	31.9	39.3	8.0	16.0
3	4.0-8.0	28.8	31.9	39.3	4.0	8.0
4	8.0-14.5	27.4	27.2	45.4	0.056	0.100
5	14.5-25.5	28.2	27.5	44.3	0.056	0.100
6	25.5-43.5	20.0	22.6	57.4	0.056	0.056
7	43.5-73.0	27.6	17.6	54.8	0.056	0.056
8	73.0-100.0	29.1	12.1	58.8	0.056	0.032

Supplementary Table 6. Simulated hydrological fluxes of the land-use options investigated, calculated using the coupled CMF – SoBraCo model. Both models are parameterised using actual field data. Identical precipitation inputs (1,892 mm y^{-1}) are assumed for all land-use systems.

Land-use Option	Evapo-transpiration [mm y^{-1}]	Interception [mm y^{-1}]	Overland flow [mm y^{-1}]	Groundwater recharge [mm y^{-1}]	Area-specific discharge [mm y^{-1}]
Abandoned pastures	928	181	75	852	927
Alnus plantations	1,597	143	38	245	283
Pinus plantations	1,410	228	29	442	471
Low-input pastures	1,186	66	75	602	677
Intense pastures	1,167	66	77	618	695

Supplementary Table 7. Soil quality indicators for the mineral top soil in each of the land-use options investigated (mean ± SD, n = 5).

**Land-use option**	**Soil depth** [cm]	**pH**	**SOC** [%]	**BS** [%]	**MBC** [mg kg^{-1}]	**C-mineralization** [g CO_2-C kg^{-1}SOC]	**N-mineralization** [mg N kg^{-1}d^{-1}]	**PO_4-P** [mg kg^{-1}]
Abandoned pastures	0-5	4.4±0.2	9.6±0.1	13.9±6.9	1,125±73	4.5±0.6	2.3±0.5	0.6±0.2
	5-10	4.5±0.2	9.3±0.7	9.2±4.8	1,050±156	3.2±0.2	2.3±0.6	0.3±0.2
Alnus plantations	0-5	4.2±0.1	8.9±1.7	37.7±6.0	1,241±215	3.5±0.2	2.7±1.2	1.4±0.6
	5-10	4.3±0.1	6.9±1.2	23.0±1.9	889±145	2.7±0.3	2.4±1.0	1.1±0.4
Pinus plantations	0-5	3.5±0.3	8.8±2.6	7.8±2.4	722±187	3.4±1.4	1.9±0.8	6.9±3.7
	5-10	3.7±0.2	4.8±0.7	5.0±2.9	429±147	4.0±0.8	1.6±0.5	4.6±1.7
Low-input pastures	0-5	4.5±0.6	11.5±1.4	19.4±3.3	1,079±169	4.2±0.6	1.0±0.5	0.8±0.5
	5-10	4.5±0.2	9.6±1.2	14.4±2.5	1,051±285	2.7±0.8	-	0.4±0.1
Intense pastures	0-5	4.0±0.1	12.8±1.1	14.8±3.0	1,281±168	3.6±0.7	3.0±2.5	11.3±7.0
	5-10	4.2±0.2	10.6±0.8	9.9±2.8	1,437±122	2.7±0.5	-	0.7±0.9

Abbreviations: SOC, soil organic carbon; BS, base saturation; MBC, carbon in microbial biomass.

Supplementary Table 8. Financial coefficients used in the evaluation of each of the land-use options investigated.

Afforesta-tion	Alternative	Time [d ha^{-1} y^{-1}]	Material [US$ ha^{-1}]	Year	Explanation
Site preparation	Both	5	30	0	Marking, distribution of plants removal of bracken; tools
Planting	*Alnus*	7	333	0	Plant density=1,111 N ha^{-1}; planting=7 d ha^{-1}; plants (incl. transport)=0.3 US$ plant^{-1}
	Pinus	7	300	0	Plant density=1,111 N ha^{-1}; planting=7 d ha^{-1}; plants (incl. transport)=0.27 US$ plant^{-1}
Infrastructure	Both	6		0	Installation of fire breaks and extraction lines
Maintenance	Both	4		1-20	Maintenance of fire breaks and extraction lines
**Production**		**Costs**	**Revenues**		
	Both	26.2 (7)	61.73 (24)	12, 16, 20	US$ m^{-3} for saw timber (fuel wood)

Pasture	Alternative	Time [d ha^{-1} y^{-1}]	Material [US$ ha^{-1}]	Year	Explanation
Site preparation	Low-input	8	20	0-1	Mechanical removal of bracken (4 applications); tools
	Intense	7	60	0	Chemical removal of bracken (3 applications); tools (herbicide =60US$ ha^{-1})
Planting	Both	45	240	0	Plant density = 32,400 N ha^{-1}; plant collection=20d ha^{-1}; plant transport =100US$ ha^{-1}; planting =25d ha^{-1};
Infrastructure	Both	1.2	84	1,6,11,16	Material and installation of fences
Maintenance	Low-input		10.4	2-20	Veterinary = 13US$ cow^{-1} y^{-1}
	Intense		20.8	2-20	Veterinary = 13US$ cow^{-1} y^{-1}
Fertilisation	Intense	4	400	0	Distribution; Fertiliser=400 US$ ha^{-1} application^{-1}
	Intense	4	73	1-20	Distribution; Fertiliser=34 US$ ha^{-1} application^{-1} (Urea 46%) + 39 US$ ha^{-1} application^{-1} (rock phosphate)
**Production**		**Costs** milking [US$ ha^{-1}]	**Revenues** meat [US$ kg^{-1}] milk [US$ l^{-1}]		
	Low-input	13	1.9/0.34	2-20	0.4 cows ha^{-1}; production period=200d ha^{-1} y^{-1}; milk quantity=4.5 l cow^{-1} d^{-1}; meat quantity=220 kg cow^{-1} y^{-1}; milking=6.3 d cow^{-1} y^{-1}
	Intense	34	1.9/0.34	1.5-20	1.1 cows ha^{-1}; production period =200d ha^{-1} y^{-1}; milk quantity=4.5 l cow^{-1} d^{-1}; meat quantity=220 kg cow^{-1} y^{-1}; milking=6.3 d cow^{-1} y^{-1}

Supplementary Table 9. Net revenues and standard deviations used in Monte Carlo simulations (CV: Coefficient of variation; SD: Standard deviation; CV and up-front net revenue from deforestation, adopted from Knoke et al.[14])

	***Alnus* plantations**			**Pinus plantations**			**Low-input pastures**			**Intense pastures**			**Pasture after forest clearing**		
**Year**	**Net revenues**	**CV**	**SD**	**Net revenues**	**CV**	**SD**	**Net revenues**	**CV**	**SD**	**Net revenues**	**CV**	**SD**	**Net revenues**	**CV**	**SD**
0	-603	0.1	±60	-570	0.1	±57	-950	0.1	±95	-1400	0.1	±140	353	0.86	±304
1	-40	0.1	±4	-40	0.1	±4	-185	0.1	±18	-95	0.1	±10	127	0.36	±46
2	-40	0.1	±4	-40	0.1	±4	85	0.36	±31	237	0.36	±85	127	0.36	±46
3	-40	0.1	±4	-40	0.1	±4	127	0.36	±46	237	0.36	±85	127	0.36	±46
4	-40	0.1	±4	-40	0.1	±4	127	0.36	±46	237	0.36	±85	127	0.36	±46
5	-40	0.1	±4	-40	0.1	±4	127	0.36	±46	237	0.36	±85	127	0.36	±46
6	-40	0.1	±4	-40	0.1	±4	31	0.36	±11	141	0.36	±51	31	0.36	±11
7	-40	0.1	±4	-40	0.1	±4	127	0.36	±46	237	0.36	±85	127	0.36	±46
8	-40	0.1	±4	-40	0.1	±4	127	0.36	±46	237	0.36	±85	127	0.36	±46
9	-40	0.1	±4	-40	0.1	±4	127	0.36	±46	237	0.36	±85	127	0.36	±46
10	-40	0.1	±4	-40	0.1	±4	127	0.36	±46	237	0.36	±85	127	0.36	±46
11	-40	0.1	±4	-40	0.1	±4	31	0.36	±11	141	0.36	±51	31	0.36	±11
12	1527	0.37	±571	1454	0.37	±538	127	0.36	±46	237	0.36	±85	127	0.36	±46
13	-40	0.1	±4	-40	0.1	±4	127	0.36	±46	237	0.36	±85	127	0.36	±46
14	-40	0.1	±4	-40	0.1	±4	127	0.36	±46	237	0.36	±85	127	0.36	±46
15	-40	0.1	±4	-40	0.1	±4	127	0.36	±46	237	0.36	±85	127	0.36	±46
16	1429	0.43	±617	1355	0.43	±583	31	0.36	±11	141	0.36	±51	31	0.36	±11
17	-40	0.1	±4	-40	0.1	±4	127	0.36	±46	237	0.36	±85	127	0.36	±46
18	-40	0.1	±4	-40	0.1	±4	127	0.36	±46	237	0.36	±85	127	0.36	±46
19	-40	0.1	±4	-40	0.1	±4	127	0.36	±46	237	0.36	±85	127	0.36	±46
20	2589	0.48	±1244	2400	0.48	±1152	127	0.36	±46	237	0.36	±85	127	0.36	±46

Supplementary Table 10. Data on the surveyed ranking of land-use options by farmers interviewed (22 Saraguro households and 37 Mestizo households); “answers” refer to number of respondents who rate an option as rank 1, 2, 3, 4, or 5

Restoration options ranked by Saraguros

	**Without subsidies**					**With subsidies**				
	**Frequency of answers ranking option with rank number:**					**Frequency of answers ranking option with rank number:**				
	**1**	**2**	**3**	**4**	**5**	**1**	**2**	**3**	**4**	**5**
Abandoned pastures	4	0	0	0	0	0	0	0	0	0
Alnus plantations	10	4	3	0	0	16	3	1	1	0
Pinus plantations	4	8	3	0	0	1	8	6	0	4
Low-input pastures	3	2	0	0	0	0	3	3	2	0
Intense pastures	1	2	3	0	1	4	4	2	4	1

Restoration options ranked by Mestizos

	**Without subsidies**					**With subsidies**				
	**Frequency of answers ranking option with rank number:**					**Frequency of answers ranking option with rank number:**				
	**1**	**2**	**3**	**4**	**5**	**1**	**2**	**3**	**4**	**5**
Abandoned pastures	5	0	0	0	0	0	0	0	0	0
Alnus plantations	13	6	3	1	0	10	6	7	2	1
Pinus plantations	5	10	5	0	0	11	6	5	0	0
Low-input pastures	5	7	4	5	0	7	7	3	2	2
Intense pastures	9	3	0	1	2	8	2	4	1	1

Supplementary Table 11. Difference between climatic and hydrologic indicator means obtained from two model runs - one with 0 and one with 10 years spin-up time. Parameterisation, forcing and indicator units are the same as those presented in Tables 4 and 5 in the main text.

Option	Evapo-transpiration	Turbulence	Overland flow	Area-specific discharge
Abandoned pastures	0	0	-2	1
Alnus plantations	0	0	2	-2
Pinus plantations	0	0	0	0
Low-input pastures	0	0	0	0
Intense pastures	0	0	0	0

Supplementary Table 12. Most important model parameters in terms of parameter uncertainty, indicated by their expected maximum deviation from mean (span ±), for each of the plants characterizing the land-use options simulated using the SoBraCo model. Table refers to parameters as described in Bendix et al.[6] and Silva et al.[46]. PDF: probability density function. NIR: Near infrared.

SoBrCoMo

Plant	Parameter	PDF Form	Mean	Span (±%)	Source of model data
Bracken, abandoned pastures	Leaf albedo NIR	Triangular	0.475	10	Göttlicher et al.[4]
	Maximum carboxylation rate		68.8		
	Quantum efficiency		0.048		Silva et al.[5]
	Root coefficient a		3		
	Root coefficient b		3		
	Leaf dimension*	Uniform	0.030	5	Destructive measurements from 1m^2 vegetation surface (n=3; values obtained: 0.025, 0.025, 0.026)
	Displacement height		0.7		
	LAI		Scenario forcing		Bendix et al.[6]
Setaria, active pastures	Leaf albedo NIR	Triangular	0.40	10	Göttlicher et al.[4]
	Maximum carboxylation rate		29.2		Silva et al.[5]
	Quantum efficiency		0.50		
	Root coefficient a		33.0		
	Root coefficient b		9		
	Leaf dimension*	Uniform	0.020	5	Destructive measurements from 1m^2 vegetation surface (n=3; values obtained: 0.022, 0.017, 0.019)
	Displacement height		0.55		
	LAI		Scenario forcing		Bendix et al.[6]
Alnus plantations	Leaf albedo NIR	Triangular	50.3	10	Göttlicher et al.[4]
	Maximum carboxylation rate		40		Oleson and Dai[7]

	Quantum efficiency		0.046		Muthuri et al.[8]
	Root coefficient a		6		Zeng[9]
	Root coefficient b		2		
	Leaf dimension*	Uniform	0.04	5	
	Displacement height		0.67		
	LAI		Scenario forcing		Cabezas-Gutierrez et al.[10]
Pinus *plantations*	Leaf albedo NIR	Triangular	43		Göttlicher et al.[4]
	Maximum carboxylation rate		37		Niinemets et al.[11]
	Quantum efficiency		0.062		
	Root coefficient a		7		Zeng[9]
	Root coefficient b		2		
	Leaf dimension*	Uniform	0.03		
	Displacement height		0.67		
	LAI		Scenario forcing		Aguirre-Salado et al.[12]

*Leaf dimension is the characteristic length of a leaf [m] in the direction of wind flow. It is used to estimate the leaf boundary layer resistance. The characteristic leaf length is hard to measure for trees, given changing wind directions and movements of flexible leaves. The default model value is 0.04 for all plant functional types from temperate needleleaf evergreen trees to C_4 grasses. Where we had no measurements we wanted to keep close to the default value, which fitted best to *Alnus*, but considered also that leaf boundary layer resistance, for a given wind speed, is lower for smaller leaf sizes in the direction of wind flow (*Pinus*).

Supplementary Table 13. Most important model parameters in terms of parameter uncertainty, indicated by span referring to the assumed minimum and maximum of the parameter (±), for CMF. PDF: probability density function.

Land-use option	Parameter	PDF	Mean	Span (±%)	Source of model data
Bracken and Setaria pastures	k_{sat} (8 soil layers)	Uniform	20, 8, 4, 0.06, 0.06, 0.06, 0.06	20	Huwe et al.[13]
Alnus and *Pinus* plantations	k_{sat} (8 soil layers)		40, 16, 8, 0.1, 0.1, 0.06, 0.06, 0.32		
All land types	Porosity (8 soil layers)		0.55, 0.55, 0.55, 0.55, 0.55, 0.51, 0.55, 0.55, 0.51, 0.51, 0.48		

Supplementary Table 14. Indicator values for the key element "Carbon relationships" in terms of uncertainty considering upper and lower 95 % confidence limits as pessimistic and optimistic estimates (rank in parentheses).

Land-use Option	**Annual biomass production** [Mg ha^{-1} yr^{-1}]			**Carbon in planta** [Mg ha^{-1}]			**Soil organic carbon** [Mg ha^{-1}]		
	Pessi-mistic	Opti-mistic	Range $P_{i\ low}$ - $P_{i\ high}$	Pessi-mistic	Opti-mistic	Range $P_{i\ low}$ - $P_{i\ high}$	Pessi-mistic	Opti-mistic	Range $P_{i\ low}$ - $P_{i\ high}$
Abandoned pastures	18.5 (2)	45.1 (2)	32-76	24.9 (2)	41.1 (1)	90-100	72.6 (5)	102.0 (5)	0
Alnus plantations	6.5 (5)	8.9 (5)	0	20.1 (3)	29.3 (4)	53-62	78.4 (3)	105.0 (3)	36-49
Pinus plantations	8.1 (4)	9.7 (4)	2-4	26.8 (1)	32.5 (3)	66-100	84.5 (1)	102.5 (4)	6-100
Low-input pastures	14.3 (3)	38.7 (3)	21-63	9.2 (5)	15.8 (5)	0	78.2 (4)	105.4 (2)	40-50
Intense pastures	43.6 (1)	56.4 (1)	100	16.5 (4)	35.2 (2)	41-77	82.1 (2)	110.5 (1)	80-100

Supplementary Table 15. Indicator values for the key element “Climate regulation” in terms of parameter uncertainty after 3,216 Monte-Carlo (MC) simulation runs considering upper and lower 95 % confidence limits as pessimistic and optimistic estimates (rank in parentheses).

**Land-use Option**	**Evapo-transpiration (ET)** [mm]			**Momentum flux** [kg m^{-1} sec^{-2}]		
	Pessimistic	Optimistic	Range $P_{i\,low}$ - $P_{i\,high}$	Pessimistic	Optimistic	Range $P_{i\,low}$ - $P_{i\,high}$
Abandoned pastures	921 (5)	936 (5)	0-0	0.0174 (5)	0.0184 (5)	0-0
Alnus plantations	1589 (1)	1600 (1)	100-100	0.2544 (2)	0.3156 (1)	86-100
Pinus plantations	1407 (2)	1415 (2)	71-73	0.2932 (1)	0.2947 (2)	93-100
Low-input pastures	1175 (3)	1197 (3)	38-39	0.0229 (4)	0.0231 (4)	2-2
Intense pastures	1157 (4)	1177 (4)	35-36	0.0252 (3)	0.0268 (3)	3-3

Supplementary Table 16. Indicator values for the key element “Hydrological regulation” (considering high area specific discharge as negative) in terms of parameter uncertainty after 3,216 Monte-Carlo (MC) simulation runs considering upper and lower 95 % confidence limits as pessimistic and optimistic estimates (rank in parentheses).

**Land-use Option**	**Overland flow** [mm y^{-1}]			**Area specific discharge** [mm y^{-1}]		
	Pessimistic	Optimistic	Range $P_{i\,low}$ - $P_{i\,high}$	Pessimistic	Optimistic	Range $P_{i\,low}$ - $P_{i\,high}$
Abandoned pastures	82 (4)	68 (3)	1-8	941 (5)	913 (5)	0-0
Alnus plantations	40 (2)	36 (2)	77-85	291 (1)	275 (1)	100-100
Pinus plantations	32 (1)	26 (1)	100-100	476 (2)	465 (2)	70-71
Low-input pastures	81 (3)	69 (4)	4-4	691 (4)	663 (4)	38-39
Intense pastures	83 (5)	71 (5)	0-0	707 (3)	683 (3)	36-36

Supplementary Table 17. Rating of the key elements “Climate regulation” in terms of uncertainty of the forcing variables due to atmospheric variability represented by a relatively wet (2005) or dry (2010) year.

Land-use option	Evapo-transpiration (ET)		Momentum flux	
	[mm] wet- dry	P_I wet - dry	[kg m^{-1} sec^{-2}] wet - dry	P_I wet – dry
Abandoned pastures	513 - 713	0 – 0	0.047 - 0.016	0 – 0
Alnus plantations	1280 – 1319	100 - 100	0.598 - 0.189	100 – 100
Pinus plantations	1026 -1146	72 – 71	0.497 - 0.157	82 – 81
Low-input pastures	957 – 974	62 – 43	0.058 - 0.020	2 – 2
Intense pastures	922 - 957	57 – 40	0.057 - 0.019	2 – 2

Supplementary Table 18. Rating of the key elements “Hydrological regulation” in terms of uncertainty in the forcing variables due to atmospheric variability represented by a relatively wet (2005) or dry (2010) year.

Land-use	Overland flow		Area-specific discharge		
Option	[mm y^{-1}] wet- dry	P_i wet- dry	[mm y^{-1}] wet- dry	$P_{i(+)}$ wet- dry	$P_{i(-)}$ wet- dry
Abandoned pastures	30 - 38	0 - 27	1345 – 859	100 – 100	0 – 0
Alnus plantations	2 -12	100 – 81	630 – 279	0 – 0	100 – 100
Pinus plantations	2 -2	99 – 100	872 – 461	34 – 28	66 - 72
Low-input pastures	24 - 52	22 – 0	915 – 583	40 – 49	60 - 51
Intense pastures	24 - 48	22 – 7	947 - 604	44 – 53	56 - 47

Supplementary Table 19. Range of index values (P_i) for the key element "Soil quality" when upper and lower 95 % confidence limits as pessimistic and optimistic estimates are used to estimate indicator values.

**Land-use option**	**pH**	**SOC** [%]	**BS** [%]	**MBC** [mg kg^{-1}]	**C-mineralization** [g CO_2-C kg^{-1}SOC]	**N-mineralization** [mg N $kg^{-1}d^{-1}$]	**PO_4-P** [mg kg^{-1}]
Abandoned pastures	93 -100	30 -72	6 - 34	59 - 71	61 - 100	33 - 100	0 - 0
Alnus plantations	73 - 98	22 - 23	100 - 100	59 - 66	0 – 33	55 - 89	11 - 19
Pinus plantations	0 - 0	0 - 0	0 - 0	0 - 0	0 – 100	26 - 66	83 – 100
Low-input pastures	92 - 100	73 - 74	42 - 46	52 - 74	26 – 57	0 - 21	0 – 2
Intense pastures	66 - 83	100 - 100	22 - 23	100-100	7 – 30	0 - 100	47 – 100

Supplementary Table 20. Indicator values for the economic key elements for an assumed discount rate of 8% in terms of uncertainty of the coefficients used in the economic calculation after 3,000 MC simulation runs considering upper and lower 95 % confidence limits as pessimistic and optimistic estimates (rank in parentheses).

	NPV (Euro/ha)			Payback period (years)		
**Land-use option**	Pessimistic	Optimistic	Range $P_{i\,low}$ - $P_{i\,high}$	Pessimistic	Optimistic	Range $P_{i\,low}$ - $P_{i\,high}$
Abandoned pastures	0 (2)	0 (5)	3-53	0 (1)	0 (1)	100-100
Alnus plantations	-153 (3)	1397 (1)	59-100	24 (3)	8 (3)	40-66
Pinus plantations	-170 (4)	1308 (2)	55-94	24 (3)	8 (3)	40-66
Low-input pastures	-409 (5)	113 (4)	0-0	40 (5)	24 (5)	0-0
Intense pastures	26 (1)	924 (3)	66-100	21 (2)	5 (2)	48-79

Supplementary Table 21. Indicator values for the "Social preference" (example without subsidy) key elements considering upper and lower 95 % confidence limits as pessimistic and optimistic estimates (rank in parentheses). Answers refer to number of respondents who rate an option as best or second best

	Answers Saraguros			Answers Mestizos		
**Land-use option**	Pessimistic	Optimistic	Range $P_{i\,low}$ - $P_{i\,high}$	Pessimistic	Optimistic	Range $P_{i\,low}$ - $P_{i\,high}$
Abandoned pastures	0 (4)	8 (4)	0-0	1 (5)	9 (5)	0-0
Alnus plantations	8 (1)	20 (1)	100-100	12 (1)	26 (1)	100-100
Pinus plantations	6 (2)	18 (2)	77-82	8 (2)	22 (2)	40-66
Low-input pastures	1 (3)	9 (3)	8-11	6 (3)	18 (3)	45-53
Intense pastures	0 (4)	8 (4)	0-0	6 (3)	18 (3)	45-53

Supplementary Table 22. t- and p-values (in parentheses) for statistical contrasts between land-use options tested in conjunction with a one-way ANOVA on rank-transformed data. Abbreviations: Ab=abandoned pastures, A=*Alnus*, P=*Pinus*, L=low-input pastures, and I=intense pastures. Standardised differences (t-values) associated with p-values <= 0.10 considered significant (indicated in bold). Contrast 1 (-4 I 1 I 1 I 1 I 1), contrast 2 (0 I 1 I 1 I -1 I -1), contrast 3 (0 I 1 I -1 I 0 I 0), and contrast 4 (0 I 0 I 0 I -1 I 1), respectively, indicate that: 1. All restoration options on average improve the ecological and socio-economic index values significantly, 2. afforestations perform significantly better than pasturing options, 3. *Alnus* does not differ significantly from *Pinus*, and 4. intense pasture is better than low-input pasture on a significance level of at least α<0.1 in most scenarios (n.s.: not significant).

	**t-values and associated p-values in parentheses for five scenarios tested:**				
**Contrast**	Scenario tested in main text	Index values weighted with relative range of maximum variation (a. in Supplementary Figure 1)	Optimistic index values (b. in Supplementary Figure 1)	Pessimistic index values (c. in Supplementary Figure 1)	Subjectively weighted index values (d. in Supplementary Figure 1)
**1. (A+P+L+I)/4 > Ab**	**2.3 (<0.025)**	**2.4 (<0.025)**	**2.6 (<0.025)**	**1.5 (<0.100)**	**1.9 (<0.050)**
**2. (A+P)/2 > (L+I)/2**	**3.1 (<0.025)**	**2.6 (<0.025)**	**2.7 (<0.025)**	**3.3 (<0.025)**	**2.4 (<0.025)**
**3. A > P**	0.9 (n.s.)	0.5 (n.s.)	0.9 (n.s.)	1.0 (n.s.)	0.7 (n.s.)
**4. L < I**	**1.6 (<0.100)**	**1.9 (<0.050)**	**1.4 (<0.100)**	**1.6 (<0.100)**	1.1 (n.s.)

Supplementary Methods

1. Background information on research area
2. Land-use options investigated
  2.1. Leaving areas abandoned
  2.2. Afforestation
  2.3. Pasture use
3. Indicators evaluated
  3.1. Ecological
    3.1.1. Biomass production and carbon-sequestration
    3.1.2. Climate and water indicators
    3.1.3. Soil quality
  3.2. Economic
  3.3. Social

1. Background information on research area

The research area is in the catchment area and valley of the Rio San Francisco (1,000-2,800 m a.s.l.) which is a deeply incised valley in the very humid eastern range "Cordillera oriental" of the Andes in southern Ecuador[15]. As such, it is representative of the biogeographical and socio-economic setting of the eastern escarpment of the tropical Andes.

Climate. Air temperature in the forest-to-pasture conversion zone of the San Francisco Valley is generally <16°C[16]. The area is characterized by very humid conditions year-round, with annual rainfall exceeding 1,800 mm (up to 6,000 mm y^{-1} including occult precipitation at 3,200 m asl) and without any pronounced dry season[17]. This is caused by a very high perennial cloud frequency of around 80%[18]. Inter-annual variability depends largely on the ENSO (El Niño / Southern Oscillation) cycle[19,20], which seems to have changed slightly since 2000[21].

Soils. Most soils in the deforestation areas are Dystrudept soils. These soils are frequently characterised by hydromorphic properties due to humid conditions, as well as by acidic pH values, enhanced soil moisture, a well-established organic layer and a poor nutrient situation (high C/N ratio, N and P deficiencies)[22-24].

Vegetation, biodiversity and land-use change effects. With the exception of the highest elevations, which are covered by subpáramo vegetation, the natural vegetation of the ridges and valleys in the study area is a slightly zonal tropical mountain rain forest[25]. The study region is considered to be one of the outstanding global hotspots of biodiversity[26]. Diversity is extraordinarily high in most species groups, for example trees (up to 37 tree species on a 20 x 20 m plot), orchids and birds[27]. World records for diversity have been found here for geometrid moths[28] and epiphytic plants[29].

Conversion of the pristine forest into agricultural areas inevitably changes the entire ecosystem and its biological diversity. *Setaria* pasture, the most common type in the study region, exhibits a low variety of accompanying herbaceous species - between 5 and 13 on average (4 m^2 minimum area), depending on the age and the intensity of management, particularly the frequency of burning[30]. The highest plant diversity of the anthropogenic systems presented here was recorded on abandoned areas, where the species-area relationship on an area of 5 x 5 m in 1999 was found to be 22.7 ± 3.4 species[31]. Ten years later, 22 to 38 species were found on plots of 10 x 10 m[31]. In contrast, on a plot of the same size (100 m^2) in the pristine forest, 125 species of vascular plants were recorded.

Diversity of 2 other groups of organisms - moths and mycorrhizae - is considered to allow for comparisons between natural forest and anthropogenic systems. Anthropogenic areas in the vicinity of the forests are found to be species sinks from the forests, mainly for the moth imagines, as even the relatively richer abandoned pastures are deficient in the fodder plants the caterpillars need. In the areas studied the species composition of the moth ensembles shifts with distance from the forest in favour of representatives of the Geometridae. The gradient in abundance of Arctiidae species is much steeper[32]. Due to the available body of data and the complexity of the subject, assessment of the biological diversity of the mycorrhizae on the variants of land use investigated is less conclusive[33,34]. High mycobiont species richness is found in the roots examined from both natural forest and afforested areas. The majority are fungal generalists which develop relationships with several plant species, including the pasture grass *Setaria sphacelata*. Up to now, the data from four tree species indicate no specificity of the mycobiont diversity in either the original or the anthropogenic ecosystems. Any differences detected between the natural forest and the abandoned

pastures are difficult to assess because they may be due purely to inherent differences in the two types of habitats, or simply to plant age.

In summary, clearing of the forest for agricultural land use causes an extraordinary decline in vascular plant and moth diversity, which may or may not be paralleled by soil fungal diversity. After areas are abandoned, plant diversity increases a little, but remains far below that of the pristine forest. Moth diversity still relies on migration rather than on the establishment of unique populations. Fungal diversity remains rich in the anthropogenic systems, particularly in afforested areas, where it is comparable to that in natural forest.

Land-use change and non-sustainable pasture management. From the valley floor upwards, the forest has been cleared by slash and burn for agricultural purposes - mostly pasture. The most common pasture grasses used are introduced species such as *Setaria sphacelata* (which is planted manually) or *Melinis minutiflora,* both of which are native to south and central Africa. In the core research area of 120 km^2, around 35% of areas that have been converted to pasture are no longer used because of infestation by weeds[35].

The nutritive value of the grasses commonly used here is rather low, and therefore, the majority of pastures are overgrazed. This sets in motion a vicious cycle, as weeds eventually overgrow the non-native pasture grasses. Bracken fern (*Pteridium arachnoideum* and *Pt. caudatum*) is the most problematic weed handicapping agriculture in tropical mountains. Fire is commonly used by the local farmers to rejuvenate pastures and remove weeds. However, tropical bracken and other aggressive weeds have proven to be fire-resistant, and therefore, in the long run, burning favours the weeds and eventually leads to abandonment of the pasture[36]. At this stage, the weeds form a closed canopy, which prevents return of the natural forest[30].

The farmers. Most of the farmers in the area are Mestizos, a term generally used to indicate people of mixed Spanish and indigenous descent. Neighbouring valleys are also inhabited by indigenous Saraguros - Quechua-speaking Indians who traditionally inhabited the Andean uplands of southern Ecuador. Farmers of both ethnic groups are colonists who arrived in the study area during the 20th century. As agro-pastoralists[37,38], they participate in both a market economy (cattle ranching for cheese, milk and meat production) and a subsistence economy (crop production, horticulture and cattle

ranching for subsistence needs). Cattle ranching is the most important market activity, and from the farmers' point of view, the most profitable. The main product drawn from cattle ranching is cheese (*quesillo* - an unsalted fresh white cheese), which is sold in the local markets. Only farmers who have good access to roads can sell milk to regional producers of dairy products. Despite both ethnic groups practicing a similar land-use system, Saraguros derive more income from cattle ranching than Mestizos and Mestizos have higher off-farm incomes (employment, remittances) than Saraguros[37,38].

Migration of farmers is an important phenomenon. According to the census of 2010, from 1,324 persons born in the study area (Imbana, Sabanilla), 335 were registered in other regions in the country and, of these, 249 lived in urban areas. Population has decreased by around 6% (from 1,807 to 1,710 people) since the previous census in 2001; also, the percentage of the population economically active in agriculture decreased from 83% in 2001 to 65% in 2010[39]. These trends are mainly due to the lack of agricultural labour in the region and the opportunity for wage labour elsewhere.

2. Land-use options investigated

2.1. Leaving areas abandoned

Abandoned areas are usually covered by bracken fern (*Pteridium arachnoideum* (Kaulf.) Maxon and *Pt. caudatum* (L.) Maxon) interspersed with quickly reproducing shrubs. Fire usually kills the above-ground parts of this vegetation, but the plants can readily re-sprout from subterranean buds, such as the short lateral rhizomes of the bracken fern. This type of fire-resistant plant community with a high potential for propagation is encountered in many abandoned farming areas of the southern Ecuadorian Andes, and can be considered a type of "novel ecosystem"[40] resulting from human activities. It forms our reference as a typical widespread ecosystem in the southern Ecuadorian Andes (phytosociological details in Hartig & Beck[30]).

2.2. Afforestation

Afforestation with native Andean alder (Alnus acuminata). This option is based on data from an afforestation experiment consisting of afforestation plots (10.8 x 10.8 m), with 6 repetitions each[41]. Bracken leaves were removed manually and saplings up to 0.5 m tall were planted. Any regrowth of bracken was removed in the same way during the following 2 years. Modelling of future growth was based on this experimental data,

using an annual mortality rate of 2 % of the number of trees per hectare and the commonly used seedling distance of 3 m x 3 m. The harvest regime began after 12 years with an initial thinning of 40% of the number of trees per hectare followed by a second thinning of the same intensity 16 years after the initial planting. The final harvest of crop trees was simulated after 20 years.

Afforestation with exotic Pinus patula. *Pinus patula* or *Eucalyptus saligna* are commonly used for afforestation in southern Ecuador. Here, we considered *Pinus patula* as a fast-growing, exotic alternative to alder. The calculations used were based on data from the previously mentioned reforestation experiment where *Pinus* was also planted, and the same protocol was used, including tree density, mortality rate and harvest regime.

2.3. Pasture

The total area of repasturisation was 4,500 m^2, and was subdivided into 4 x 4 m plots. Each treatment was investigated in 4 parallels to average possible variations in site quality. The procedures used for weed removal and planting of grass are described in Roos et al.[42].

Weed removal and low-input pasture. Pasture rehabilitation[42] proved successful when three consecutive steps were followed: i) Bracken control by repetitive cutting of the leaves during 1 year. Four such cutting campaigns were sufficient. ii) After a short fallow period, the common pasture grass, *Setaria sphacelata,* was purchased from local farmers and planted manually at the customary density (32,400 plantlets x ha^{-1}). Planting of 1 ha required approximately 1 month. After approximately one and half years, the grass had reached a cover percentage of more than 70% and grazing could begin. iii) Grazing was simulated by cutting the grass to a residual height of 20 cm. Regrown bracken fronds were kept in check by simulating trampling by cattle. Two grazing rounds per year were thought to represent a compromise between sustainable and maximal grass yields, and were expected to result in sufficient trampling to prevent recovery of bracken.

Weed removal and intense pasture use. This option paralleled low-input pasture, but bracken removal was accomplished by spraying a common herbicide (“Combo”: picloram: 960 g a.i. ha^{-1} and metsulfuron methyl: 2,400 g a.i. ha^{-1}; Dow Agro Science)

three times (within nine months) and subsequent application of chemical fertiliser. The results obtained during two years of pasture fertilisation and grazing revealed co-limitation of productivity by levels of both N and P, with the latter being particularly important. Fertilisation with NPK (150 kg N, 86 kg P, and 107 kg K ha^{-1} y^{-1}) did not change the nutritive value of the pasture grass, but increased its growth and biomass production significantly (Supplementary Table 1). Three grazing rounds per year were simulated, with a higher number of cattle than in the low-input option to insure sufficient trampling of the fern. High yields have been achieved on long-standing pasture on favourable sites using a fertilisation protocol to replace the nutrients removed by grazing (50 kg N and 10 kg P ha^{-1} y^{-1}; 1.25-fold increase from 9.02 Mg DM ha^{-1} y^{-1} to 11.2 Mg DM ha^{-1} y^{-1})[1,43]. In these cases, the levels of protein, N, P and Ca were also much higher than in our research pastures (Supplementary Table 1). According to the National Research Council[44], about 0.89 kg crude protein per day is required by highly productive mature dairy cattle. The daily requirements of cattle for P (10 g to 28 g) and Ca (14 g to 56 g) vary strongly depending on such factors as breed, current body weight and lactation status. In the research area, only a small percentage of the cattle are dairy cows, and breeding of calves is very common. Therefore the number of possible cattle is higher than calculated from the nutritive value assumed to be necessary for dairy cattle. For the low-input pasture management option modelled here, 0.4 head of cattle per hectare were assumed, and for the intense pasture, 1.1 head. These are still modest assumptions, because on high yielding pasture twice as many cattle could be maintained.

3. Indicators evaluated

3.1. Ecological

3.1.1. Biomass production and carbon sequestration

The data used for the evaluation of the carbon relationships of the 5 land-use options are presented in Supplementary Tables 2 and 3. Average carbon stocks ("Carbon in planta") are calculated individually for above- and below-ground biomass (Supplementary Table 3) and average standing crop data, which is common practice in carbon assessment for of ecosystems[45]. Total carbon stocks in the above- and below-ground biomass, and in the top 20 cm soil layer are used to assess the carbon balances of the various land-use options. When equilibria are considered (abandoned area, pasture), constant carbon stocks are assumed to take into account smaller fluctuations

due to weather conditions[46]. In the afforestation options, carbon stocks increase with tree growth but decrease after thinning.

Abandoned areas. Bracken leaf is assumed to have an average lifetime of 8 months[47]. Thus, annual aboveground biomass production is estimated as standing crop volume times 12/8, and is equal to 9.15 Mg ha^{-1} y^{-1}. Because the lifetime of the below-ground bracken organs (rhizomes and roots) is difficult to measure, an average value of 22.6 Mg ha^{-1} y^{-1} for bracken below-ground biomass production is estimated using the SoBraCo-Model[46].

Pinus and Alnus plantations. Volume equations obtained from the literature are used to calculate both the standing and the commercial timber volume for *A. acuminata*[48] and *P. patula*[49]. Based on the number of trees per hectare (*N)*, the diameter at 1.3 m height (*dbh*), and the mean total height (*ht), e*stimated regression curves are as follows:

$dbh_{Alnus} = 1.516 – 1.778 \ln(N) + 12.287 \ln(age)$

$dbh_{Pinus} = 1.803 – 0.969 \ln(N) + 10.165 \ln(age)$

$ht_{Alnus} = EXP(5.132 + 0.644 \ln(dbh))$

$ht_{Pinus} = 1282.369 + 249.983 \ln(dbh) – 214.119 \ln(N) + 273.475 \ln(age)$;

dbh = mean diameter at breast height [cm], *N* = number of trees/hectare, *age* = age of plantation [years], ht = mean height of trees [cm].

Above-ground biomass is calculated using the equation developed by Figueroa-Navarro et al.[50] for *P. patula* and that from Acosta-Mireles et al.[3] for *A. acuminata*. Below-ground biomass is estimated based on a shoot : root ratio of 1:0.205[2]. The concentration of carbon for both species in litter and in timber is around 50 %. Therefore, the rate of carbon sequestration is calculated as half of the corresponding biomass. For the leaf area indices (LAI) methods used, see Materials and Methods, chapter Climate. The resulting stand characteristics for afforestation with *A. acuminata* and *P. patula* are shown in Supplementary Table 4. These figures take both thinnings and mortality into account.

Low-input and intense pasture. In the grazing simulation, above-ground biomass was harvested and both fresh and dry weights determined. This material is included in the calculation of annual biomass production. Below-ground biomass production is calculated using the SoBraCo-model parameterised with adjusted LAI and vegetation height data collected during the second management year. Average standing above-

ground crop is calculated, accounting for the material extracted by simulated grazing. Grass biomass from 0 to 20 cm in height is added to this amount to account for the material not removed in the grazing simulation. Below-ground average standing crop was analysed from soil cores, as described in *Material and Methods*. Uncertainty from using 95 % confidence limits of indicators to estimate indices is reported in Supplementary Table 5.

3.1.2. Climate and water indicators

Comprehensive validation of the SVAT and vegetation growth model CLM (Community Land Model), which is the numerical basis of SoBraCoMo[46], reveals that the uncertainty of GPP and ET simulations for all plant functional types lies well within the uncertainty of FLUXNET - based validation estimates (e.g. Bonan et al.[51]). In the current study, the simulations are forced using data from an average meteorological year (2008) as measured at a specifically designed micrometeorological station onsite (lat.: 3.96427°S, long: 79.07689°W, 2109 m a.s.l.)[46]. The measurements used are solar irradiation, air temperature (0.5 and 2 m), relative humidity (0.5 and 2 m), leaf temperature (sunlit and shaded), wind speed, rainfall, soil water content and soil temperature. The year 2008 was selected as a typical year for the area based on data collected between 1981 and 2010 at the INAMHI (Ecuadorian Weather Service) station Loja La Argelia (lat.: 4.03055°S, long: 79.19944°W, 2160 m a.s.l.). This is the only long-term data set available (temperature and rainfall only) for the wider study area. Average temperature and monthly total rainfall for 2008 (15.9°C, 115 mm/month) are similar to long-term averages (16.0°C with SD of ±0.8°C; 78.1 mm/month with SD of ±48.9 mm/month). The annual time series used for model forcing consists of hourly aggregated meteorological variables and is applied repeatedly for the entire 20-year period.

Required plant-specific model parameters are derived either from measurements at leaf and root levels in the study site, or from data available from the literature (for further details refer to Silva et al.[46], Supplementary Table 12 and Potthast et al.[52] for soil parameters). The parameters derived are leaf area index, leaf tilt angle, leaf reflectance and transmittance, quantum yield, carboxylation rate of sunlit/shade leaves, Q_{10} temperature coefficient, entropy factor, deactivation energy, dark respiration at 25°C of sunlit/shaded leaves, activation energy for respiration, vertical density of root distribution, root/rhizome biomass, root/rhizome C:N ratio and CO_2 to biomass conversion factor.

In the coupled model framework (see next para), the SoBraCoMo calculates the main water and momentum fluxes between the canopy and the atmosphere, which are important for assessing land-use change effects on climate regulation functions. The variables considered for the climatic assessment are (i) evapotranspiration and (ii) turbulence production, with the latter expressed as the sum of zonal and meridional momentum fluxes. With regard to carbon and biomass estimates, the model also computes net canopy photosynthesis, based on the “two-big-leaf” approach. Validation with porometer observations showed a high level of accuracy for the model results, with deviations between simulated and observed leaf net assimilation of less than 5%[46].

The Catchment Modelling Framework (CMF[53]) is a state-of-the-art programming library used to create hydrological models which are highly modular and connectible to other models. Due to its modular structure, it is suitable for simulating a wide variety of hydrological conditions and has proven to provide reliable results in complex, intrinsically coupled modelling systems[54,55]. Eight soil layers of increasing thicknesses from the top downwards are summed to reach a total column depth of 1 m (Supplementary Table 5). The movement of soil water within each layer is simulated using the Richards equation. Water leaving the soil column is directed to the ground water using a Dirichlet boundary condition with a constant negative pressure. Slope in the study area is considered using a hydraulic gradient suitable for a slope of 10%.

It should be stressed that CMF is directly coupled with the SoBraCo-model using a Python interface[56]. Atmospheric variables are computed using SoBraCoMo, while soil and surface hydrology are determined using CMF. The setup described in the main section, the discretisation of the 1D soil column and the soil properties are all presented in Supplementary Table 5. The water fluxes generated by the model and presented in Supplementary Table 6 account for more than 98% of the incoming precipitation (all intercepted water evaporates, and is, therefore, included in the evapotranspiration figures). The remainder (less than 2%) can be attributed to storage changes and minor rounding errors.

The spin-up time of the coupled SoBraCo-CMF model was initially set to 0. However, because some previous work[57] has shown a potential influence of the spin-up time on model results, we compare the results from runs using 0 and 10 years of spin-up time. The outcome of this comparison presented in Supplementary Table 11 shows either no

(climatic indicators) or non-relevant (hydrologic indicators) differences in the model outputs.

Model output uncertainty analysis of the coupled SoBraCo-CMF model framework with regard to parameter uncertainty is conducted using the Monte Carlo (MC) technique, as proposed by Veerbeeck et al.[58] for SVAT-type models.

Parameter uncertainty analysis is normally based on a model sensitivity study to unveil the model parameters to which the model output is most sensitive. A one-at-a-time sensitivity analysis of the CLM model for the study area[59] revealed sensitive model parameters similar to those found for the FORUG model evaluated by Veerbeck et al.[58]. Based on this evaluation, the parameters displayed in Supplementary Table 12 are used in the parameter uncertainty analysis conducted in this study. Most are related to photosynthesis and plant water regulation. The two most sensitive CMF parameters - porosity and saturated hydrologic conductivity (k_{sat}) - are shown in Supplementary Table 13. The form of the probability density functions (PDFs) of the model parameters and the percentage deviation from the mean used for the MC analysis are chosen as either uniform or triangular, based on analysis of the field data and the PDF types used by Veerbeeck et al.[58]. The mean values of the parameters are derived from sources indicated in Supplementary Tables 12 and 13.

It should be stressed that one of the most sensitive parameters - the Leaf Area Index (LAI) – is a forcing parameter in our study which is allowed to change over time based on the individual management story lines of each of our land use options. While mean LAI is generally based on observational data in the field (refer to Supplementary Table 12), here it is modified according to activities undertaken during the 20-year model run in a particular management option (e.g. timber extraction, grazing), as defined by the requirements of the economic assessment (Supplementary Fig. 1). For tree plantations, the LAI scenarios are certainly very conservative. In the first years, when trees have not yet formed a closed canopy (achieved after 3 to 6 years), no LAI and thus, no production of biomass is modelled, although production levels similar to those in the abandoned pastures are to be expected. We are aware that this leads to an underestimation of the performance of the afforestation areas, but accept this. Thus, the better performances obtained for the afforestation options represent conservative estimates and underline the robustness of our assessment. In the MC analysis, the PDF form and relative deviation (in %), shown in Supplementary Table 13 are applied to the

respective mean LAI scenario value at each time step t as shown in Supplementary Fig. 1.

To properly analyse the output variance of the model, we include more than 3,000 simulation runs into the MC analysis. To analyse the robustness of the assessment scheme, the SEM output of all simulation runs is extracted and the 95 % confidence limits are computed for the climatic and hydrological indicators, to calculate the pessimistic and optimistic scenarios. The results for the climate indicators point to a general robustness of the assessment scheme. Only for the indicator momentum flux, *Alnus* and *Pinus* change rank position, but by leaving the afforestation options in the two top positions (Supplementary Table 15). With regard to the hydrological ratings, small changes in the overland flow are observed (Supplementary Table 16). While the first and second ranks remained the same for *Pinus* and *Alnus*, changes occur only in one case for the indicator overland flow which is characterized by very small differences in mean values. Here, the third best option is now low-input pasture changing rank position with abandoned pasture. However, no change is found for the area-specific discharge. The integrated P_k rankings do neither change for the “less is better” option nor for the “more is better option”.

To assess the uncertainty of the final model output, given input (forcing) variable uncertainty with regard to atmospheric variability, we simulate the two most extreme meteorological years observed in the study area between 1998 and 2011 - the relatively dry year in 2010 (annual total of rainfall 1582 mm; anomaly = -338 mm = -1.8 SEM) and the wet year in 2005 (annual total of rainfall 2135 mm; anomaly = +165 mm = +0.9 SEM). Considering the simulated climatic indicators for our assessment scheme (Supplementary Table 17) the simulations during relatively dry and wet years do not change the integrated ranking P_k based on the climate indicators. With regard to the hydrological indicators, no relevant shift in the integrated assessment for either the “more is better” or the “less is better” option becomes visible (Supplementary Table 18). Only the low-input and intense pasture options changed rank position.

In summary, the integrated assessment scheme for climatic and hydrological indicators proves to be very robust against parameter uncertainties and oscillations in forcing variables.

3.1.3. Soil quality

The soil quality indicators for plant growth presented in Supplementary Table 7 are well known I) to vary in response to land-use change[43], II) to support plant productivity[60] and III) to contribute to soil biodiversity[61]. The variation of indices when using 95 % confidence limits is reported in Supplementary Table 19.

3.2. Economic

We use state-of-the-art methods - net present value (NPV) and payback periods - to evaluate the economic attractiveness of each of the land-use options[62], as well as the cost:benefit ratios of nature conservation[63]. The net present values (NPV_i) and payback periods (PBT_i) for each land-use option are computed as follows:

$$
\begin{aligned}
&NPV_i = \sum_{t=0}^{T} r_{i,t} \cdot q^{-t} \\
&PBT_i = t^* \\
&\text{subject to} \\
&\sum_{t=0}^{t^*} r_{i,t} \cdot q^{-t} = 0 \\
&q = (1 + \frac{d}{100}) \\
&t \in T(0,1,...,20)
\end{aligned}
\qquad (1)
$$

where t is a point in time; T is the period of 20 years; $r_{i,t}$ is the net revenue of option, i, at time, t; q is the discount factor, d is the discount rate (either 5 or 8 %); and t^* indicates the payback period, defined as the time needed until cumulative discounted net revenues cover the up-front costs.

The results obtained are a good fit to the range of values achieved in other studies[64]. We concentrate on provisioning services when investigating the potential value of a land-use option, as financial consequences are crucial drivers of land-use decisions[65]. As labour plays an important role in both forestry and agriculture, a daily wage of 10 US$ is assumed. In the case of *afforestation*, the harvestable volume (m^3 ha^{-1} without bark) of commercial and non-commercial timber is the production quantity of saw timber and fuel wood respectively (Supplementary Table 8). Costs for site preparation and planting are also taken into account (603 US$ ha^{-1} for *A. acuminata,* and 570 US$ ha^{-1} for *P. patula*). These costs include marking, distribution of saplings, removal of bracken, seedling prices (*A. acuminata*: 0.3 US$; *P. patula*: 0.27 US$ per seedling), labour used

for planting, and tools. In addition, expenses for the installation (60 US$ ha^{-1}) and annual maintenance (40 US$ ha^{-1}) of fire breaks and extraction lines are considered. Revenues minus these costs are calculated with respect to both the quantity and quality of timber extracted (costs: 26.7 US$ m^{-3} for saw timber and 7 US$ m^{-3} for fuel wood; revenues: 61.73 US$ m^{-3} for saw timber and 24 US$ m^{-3} for fuel wood; Supplementary Table 8).

For *pasture*, economic data originate either from field experiments, from household surveys (Supplementary Table 8), or from the Statistics homepage of the Food and Agriculture Organization (faostat.fao.orgs). The production of milk and meat is based on the number of cows per hectare (low-input: 0.4 cows ha^{-1}; intense: 1.1 cows ha^{-1}), and allows for a minimum pasture preparation phase of either 24 (low input pasture use) or 18 (intense pasture) months. Expenses for site preparation and planting sum to 950 US$ ha^{-1} for low-input and 1,400 US$ ha^{-1} for intense pasture. Costs included in these figures are labour for mechanical (low-input) or chemical (intense) bracken removal, tools, purchase, transport and planting of mother tussocks (32,400 ha^{-1}) and, in the case of intense pasture, fertilisation. While the purchase price of cows is excluded from the expenditures considered, costs for infrastructure such as fencing (year 1; 6; 11; 16), as well as expenses for veterinary care (13 US$ cow^{-1} y^{-1}) are also calculated. Assuming a proportion of cows for milk production and cows for meat production of 1:1, production costs (costs for milking: 13 US$ ha^{-1} for low-input and 34 US$ ha^{-1} for intense pasture) versus revenues (0.34 US$ l^{-1} for milk and 1.9 US$ kg^{-1} for meat are calculated (Supplementary Table 8). Finally, discounted returns (5% and 8%) and payback periods are calculated for all options.

To obtain a reference scenario, we also simulate the economic coefficients of the currently prevailing form of land-use in the study region, which is low-input pasture after forest clearing (BAU scenario). Here, farmers clear forest areas up front and may sell at least part of the standing timber. According to Knoke et al.[14]. 42.6 cubic meters of merchantable timber (dbh larger than 40 cms) can be expected from a typical forest in the study area, of which 50% remains after processing. After subtracting harvesting costs and expenses for pasture establishment, the farmer still receives positive net revenues of US$ 378 ha^{-1} (Supplementary Table 9). From year 2 onwards, the same net revenues are considered as those assumed for low-input pasture.

The coefficients used for the Monte-Carlo simulations are adopted from Knoke et al.[14] and are presented in Supplementary Table 9 (see also Material and Methods in main text). The impact of uncertainty on the index values is contained in Supplementary Table 20.

3.3. Social

We performed preference analysis - a standard interview method used to quantify farmers' subjective valuation of various land-use alternatives[66-68]. Our results show that farmers' preferences are influenced by characteristics of both the farm and the farmer and by the personal costs and benefits that farmers expect. The interviews took place in October and November of 2011 in El Tibio (Saraguro community), Los Guabos (Mestizo community), and along the road known as Loja-Zamora (scattered Mestizo farms). Of the 60 interviews, 59 could be used for the ranking procedure, 37 of which were with Mestizo and 22 with Saraguro farmers. The preferences are ranked from 1 (best option) to 5 (lowest rank). Two scenarios are then tested - one in which farmers rehabilitate the abandoned areas using their own means (without subsidies) and a second in which farmers receive financial support for major inputs (e.g. seedlings, fertiliser, labour) from external agencies (with subsidies). Performance indices (P_i) are calculated from R_i values which represent the total number of responses indicating a specific option as either the best or the second best alternative (see Table 8 in main text). For the variation of indices under uncertainty refer to Supplementary Table 21.

Supplementary References

1. Potthast, K., Hamer, U. & Makeschin, F. Land-use change in a tropical mountain rainforest region of southern Ecuador affects soil microorganisms and nutrient cycling. *Biogeochemistry* **111,** 151-167 (2012).
2. Mokany, K., Raison, R. J. & Prokushkin, A. S. Critical analysis of root : shoot ratios in terrestrial biomes. *Glob. Change Biol.* **12,** 84-96 (2006).
3. Acosta-Mireles, M., *et al.* Aboveground biomass estimation by means of allometric relationships in six hardwood species in Oaxaca, Mexico. *Agrociencia* **36,** 725-736 (2002).
4. Göttlicher, D., Albert, J., Nauß, T. & Bendix, J. Optical properties of selected plants from a tropical mountain ecosystem - Traits for Plant Functional Types to parameterize a land surface model. *Ecol. Model.* **222**, 493-502 (2011).
5. Silva B., et al. in: *Ecosystem Services, Biodiversity and Environmental Change in a Tropical Mountain Ecosystem of South Ecuador* (eds Bendix, J., et al.) Ecological Studies 221, 331-342 (Springer, 2013).
6. Bendix, J., *et al.* Model parameterization to simulate and compare the PAR absorption potential of two competing plant species. *Int. J. Biometeorol.* **54**, 283-295 (2010).
7. Oleson, K., Y. Dai, *et al.* Technical Description of the Community Land Model (CLM). NCAR Technical Note NCAR/TN-461+STR, DOI: 10.5065/D6N877R0 (2004).
8. Muthuri, C.W., Ong, C.K., Black, C.R., Ngumi, V.W. & Mati, B.M. Tree and crop productivity in Grevillea, Alnus and Paulownia-based agroforestry systems in semi-arid Kenya. *For, Ecol. Manage.* **212**, 23–39 (2005)
9. Zeng, X. Global vegetation root distribution for land modeling. *J. Hydrometeor.* **2**, 525-530 (2001).
10. Cabezas-Gutierrez, M., *et al.* Un modelo para la estimación del área foliar en tres especies forestales de forma no destructiva. *Colombia Revista U.D.C.A Actualidad & Divulgación Científica* **12,**131-140 (2009).
11. Niinemets, U., Lukjanova, A., Turnbull, M. H. & Sparrow, A. D. Plasticity in mesophyll volume fraction modulates light-acclimation in needle photosynthesis in two pines. *Tree Physiol.* **27**, 1137-1151 (2007).
12. Aguirre-Salado, C.A., *et al.* Mapping Leaf Area Index and canopy cover using hemispherical photography and spot 5 HGR data regression and K-NN. *Agrociencia* **45,**105-119 (2011).
13. Huwe, B., Zimmermann, B., Zeilinger, J., Quizhpe, M. & Elsenbeer, H. in *Gradients in a Tropical Mountain Ecosystem in Ecuador* (eds Beck, E., Bendix, J., Kottke, I., Makeschin, F., & Mosandl, R.) Ecological Studies 198, 375-386 (Springer, 2008).
14. Knoke, T., *et al.* Effectiveness and distributional impacts of payments for reduced carbon emissions from deforestation. *Erdkunde* **63**, 365-384 (2009).
15. Bendix, J. & Beck, E. Spatial aspects of ecosystem research in a biodiversity hot spot of southern Ecuador – an introduction. *Erdkunde* **63,** 305-308 (2009).

16. Bendix. J., *et al.* in *Gradients in a Tropical Mountain Ecosystem in Ecuador* (eds Beck, E., Bendix, J., Kottke, I., Makeschin, F., & Mosandl, R.) Ecological Studies 198, 281-290 (Springer, 2008).

17. Rollenbeck, R. & Bendix, J. Rainfall distribution in the Andes of southern Ecuador derived from blending weather radar data and meteorological field observations. *Atmos. Res.* **99,** 277–289 (2011).

18. Bendix, J., Rollenbeck, R., Göttlicher, D. & Cermak, J. Cloud occurrence and cloud properties in Ecuador. *Clim. Res.* **30,** 133-147 (2006).

19. Bendix, J. Precipitation dynamics in Ecuador and Northern Peru during the 1991/92 El Niño - A Remote Sensing perspective. *Int. J. Remote Sens.* **21,** 533-548 (2000a).

20. Bendix, J. A comparative analysis of the major El Niño events in Ecuador and Peru over the last two decades. *Zbl. Geol. Paläontol.* (Teil I. H. 7/8), 1119-1131 (2000b).

21. Bendix, J. *et al.* El Niño meets La Niña – anomalous rainfall patterns in the "traditional" El Niño region of southern Ecuador. *Erdkunde* **65,** 151-167 (2011).

22. Schrumpf, M., Guggenberger, G., Valarezo, C. & Zech, W. Tropical montane rain forest soils. Development and nutrient status along an altitudinal gradient in the southern Ecuadorian Andes. *Erde* **132,** 43–59 (2001).

23. Iost, S., Makeschin, F., Abiy, M. & Haubrich, F. in *Gradients in a Tropical Mountain Ecosystem in Ecuador* (eds Beck, E., Bendix, J., Kottke, I., Makeschin, F., Mosandl, R.) Ecological Studies 198, 217-228 (Springer, 2008).

24. Moser, G., Röderstein, M., Soethe, N., Hertel, D. & Leuschner, C. in *Gradients in a Tropical Mountain Ecosystem in Ecuador* (eds Beck, E., Bendix, J., Kottke, I., Makeschin, F., & Mosandl, R.) Ecological Studies 198, 229-242 (Springer, 2008).

25. Homeier, J., Werner, F. A., Gradstein, S. R., Breckle, S.-W. & Richter, M. in *Gradients in a Tropical Mountain Ecosystem in Ecuador* (eds Beck, E., Bendix, J., Kottke, I., Makeschin, F., & Mosandl, R.) Ecological Studies 198, 87-100 (Springer, 2008).

26. Barthlott, W., Lauer, W. & Placke, A. Global Distribution of Species Diversity in vascular Plants: Towards a World map of phytodiversity. *Erdkunde* **50,** 317-328 (1996).

27. Liede-Schumann, S. & Breckle, S.-W. *Provisional Checklists of flora and fauna of the San Francisco Valleys and its surroundings*. Ecotropical Monographs, No. 4 Society of Tropical Ecology (2008).

28. Brehm G. & Fiedler, K. Diversity and community structure of geometrid moths of disturbed habitat in a montane area in the Ecuadorian Andes. *J Res Lepidoptera* **38,** 1-14 (2005).

29. Werner, F. A., Homeier, J. & Gradstein, S. Diversity of vascular epiphytes on isolated remnant trees in the montane forest belt of southern Ecuador. *Ecotropica* **11,** 21-40 (2005).

30. Hartig, K. & Beck, E. The bracken fern (Pteridium arachnoideum (Kaulf.) Maxon) dilemma in the Andes of Southern Ecuador. *Ecotropica* **9,** 3-13 (2003).

31. Homeier, J., *et al.* in *Ecosystem Services, Biodiversity and Environmental Change in a Tropical Mountain Ecosystem of South Ecuador* (eds Bendix, J., et al.) Ecological Studies 221, 93-106 (Springer, 2013).

32. Fiedler, K., Hilt, N., Brehm, G. & Schulze, C. H. in *Stability of Tropical Rain Forest Margins* (eds Tscharntke, T., Leuschner, C., Zeller, M., Guhardja, E. & Didin, A.) 39-60 (Springer, 2007).

33. Haug, I., *et al.* Species-rich but distinct arbuscular mycorrhizal communities in reforestation plots on degraded pastures and in neighboring pristine tropical mountain forest. *Trop. Ecol.* **51,** 125-148 (2010).

34. Haug, I., Setaro, S. & Suárez, J. P. Reforestation sites show similar and nested AMF communities to an adjacent pristine forest in a tropical mountain area of South Ecuador. *PLOS ONE* 8, DOI: 10.1371/journal.pone.0063524 (2013).

35. Curatola Fernández, G. F., Silva, B., Gawlik, J., Thies, B. & Bendix, J. Bracken fern frond status classification in the Andes of southern Ecuador: combining multispectral satellite data and field spectroscopy. *Int. J. Remote Sens.* **34,** 7020-7037 (2013).

36. Beck, E., et al. in *Ecosystem Services, Biodiversity and Environmental Change in a Tropical Mountain Ecosystem of South Ecuador* (eds Bendix, J., et al.) Ecological Studies 221, 355-370 (Springer, 2013).

37. Pohle, P., Gerique, A., Park, M. & López Sandoval, M. F. in: *Tropical Rainforests and Agroforests Under Global Change* (eds Tscharntke, T., et al.) 477-503 (Springer , 2010).

38. Pohle, P., Gerique, A., López, M.F. & Spohner, R. in *Ecosystem Services, Biodiversity and Environmental Change in a Tropical Mountain Ecosystem of South Ecuador* (eds Bendix, J., et al.) Ecological Studies 221, 219-234 (Springer, 2013).

39. INEC-REDATAM. Censo de Población y Vivienda. Sistema Integrado de Consultas [Census of population and housing. Integrated system of queries]. Retrieved from http://redatam.inec.gob.ec/cgibin/RpWebEngine.exe/PortalAction?&MODE=MAIN&BASE=CPV2010&MAIN=WebServerMain.inl (10.11.2013). Spanish (2001-2010).

40. Ellis, E. C. Anthropogenic transformation of the terrestrial biosphere. *Phil. Trans. R. Soc. A* **369,** 1010-1035 (2011).

41. Aguirre, N., Palomeque, X., Weber, M., Stimm, B., Günter, S. in *Silviculture in the Tropics* (eds Günter, S., Weber, M., Stimm, B. & Mosandl, R.) Tropical Forestry 8, 513-524 (Springer, 2011).

42. Roos, K., Rödel, H. G. & Beck, E. Short- and long-term effects of weed control on pastures infested with *Pteridium arachnoideum* and an attempt to regenerate abandoned pastures in South Ecuador. *Weed Res.* **51,** 165-176 (2011).

43. Hamer, U., Potthast, K., Burneo, J. & Makeschin, F. Nutrient stocks and phosphorus fractions in mountain soils of Southern Ecuador after conversion of forest to pasture. *Biogeochemistry* **112,** 495-510 (2013).

44. National Research Council. *Nutrient Requirements of Dairy Cattle*. Washington, DC: The National Academies Press (2001).

45. Masera, O. R., *et al.* Modeling carbon sequestration in afforestation, agroforestry and forest management projects: the CO2FIX V.2 approach. *Ecol. Model.* **164,** 177-199 (2003).

46. Silva, B. S. G., *et al.* Simulating canopy photosynthesis for two competing species of an anthropogenic grassland community in the Andes of southern Ecuador. *Ecol. Mod.* **239,** 14–26 (2012).

47. Roos, K., Rollenbeck, R., Peters, T., Bendix, J. & Beck, E. Growth of tropical bracken (Pteridium arachnoideum): Response to weather variations and burning. *Invasive Plant Sci. Manage.* **3,** 402-411 (2010).

48. Segura, M., et al. Respuesta a la fertilización con P en plantaciones de Jaúl (*Alnus acuminata*) en andisoles de la cuenca del río Virilla, Costa Rica [Effect of P fertilization on the growth of *Alnus acuminata* plantations at Andisols of the Virilla river watershed, Costa Rica]. *Agronomia Costarricense* **29,** 121-134. Spanish (2005).

49. Valdez Lazalde, J.R. & Lynch, D. K. Merchantable and total volume equations for thinned natural stands of Patula Pine in Puebla, Mexico. *Agrociencia* **34,** 747-758 (2000).

50. Figueroa-Navarro, C. M., *et al.* Biomass estimation on a managed Pinus patula Schltdl. et Cham. forest at Zacualtipan, Hidalgo State. *Revista Mexicana de Ciencias Forestales* **1,** 95-104 (2010).

51. Bonan, G. B., *et al.* Improving canopy processes in the Community Land Model version 4 (CLM4) using global flux fields empirically inferred from FLUXNET data. *J. Geophys. Res.* **116**, G02014, doi:10.1029/2010JG001593 (2011).

52. Potthast, K., Hamer, U., Makeschin, F. Impact of litter quality on mineralization processes in managed and abandoned pasture soils in Southern Ecuador. *Soil Biol. Biochem.* **42**, 56–64 (2010).

53. Kraft, P., Vaché, K. B., Frede, H.-G. & Breuer, L. CMF: A Hydrological Programming Language Extension For Integrated Catchment Models. *Environmental Modelling & Software* **26**, 828-830 (2011).

54. Kraft, P., Multsch, S., Vaché, K., Frede, H.-G. & Breuer, L. Using Python as a coupling platform for integrated catchment models. *Adv. Geosci.* **27,** 51–56 (2010).

55. Haas, E., *et al.* LandscapeDNDC: a process model for simulation of biosphere–atmosphere–hydrosphere exchange processes at site and regional scale. *Landsc. Ecol.* **28,** 615-636 (2013).

56. Windhorst, D., *et al.* in *Ecosystem Services, Biodiversity and Environmental Change in a Tropical Mountain Ecosystem of South Ecuador* (eds Bendix, J., et al.) Ecological Studies 221, 275-286 (Springer, 2013).

57. Yang, Z.-L., Dickinson, R.E., Henderson-Sellers, A. & Pitman, A.J. Preliminary study of spin-up processes in land surface models with the first stage data of project for intercomparisons of land-surface parameterization schemes phase 1(a). *J. Geophys. Res.* D8 100, 16, 553-578 (1995).

58. Veerbeck, H., Samson, R., Verdonck, F. & Lemeur, R. Parameter sensitivity and uncertainty of the forest carbon flux model FORUG: a Monte Carlo analysis. *Tree Physiology* **26**, 807–817 (2006).

59. Göttlicher, D. Plant functional types for land surface modelling in South Ecuador - Spatial delineation, sensitivity and parameter determination. PhD thesis, Faculty of Geography,

University of Marburg, 123 pp (2010). (http://archiv.ub.uni-marburg.de/diss/z2011/0061/, access: 13 July 2014).

60. Andrews, S. S., Karlen, D. L. & Cambardella, C. A. The soil management assessment framework: A quantitative soil quality evaluation method. *Soil Sci. Soc. Am. J.* **68,** 1945-1962 (2004).

61. Pulleman, M., et al. Soil biodiversity, biological indicators and soil ecosystem services - an overview of European approaches. *Curr. Opin. Environ. Sustain* **4,** 529-538 (2012).

62. Cubbage, F., et al. Investment returns for selected plantation and native forests in South America and the Southern United States. *New. For.* **33,** 237–255 (2007).

63. Balmford, A., et al. Economic reasons for conserving wild nature. *Science* **297**, 950-953 (2002).

64. Knoke, T., Steinbeis, O. E., Bösch, M., Román Cuesta, R. M. & Burkhardt, T. Cost-effective compensation to avoid carbon emissions from forest loss: An approach to consider price–quantity effects and risk-aversion. *Ecol. Econ.* **70,** 1139-1153 (2011).

65. Lambin, E. F., et al. The causes of land-use and land-cover change: moving beyond the myths. *Glob. Environ. Change* **11**, 261-269 (2001).

66. Batz, F.-J., Janssen, W. & Peters, K. J. Predicting technology adoption to improve research priority-setting. *Agricult Econ.* **28,** 151-164 (2003).

67. Bekele, W. Analysis of Farmers' Preferences for Development Intervention Programs: A Case Study of Subsistence Farmers from East Ethiopian Highlands. *Afr. Dev. Rev.* **18,** 183-204 (2006).

68. Hernández-Hernández, R. M., et al. La integración del conocimiento local y científico en el manejo sostenible de suelos en agroecosistemas de sabanas. (The integration of local and scientific knowledge in the sustainable management of soils in savannah agroecosystems). *Interciencia* **36,** 104-112 (2011).
